# Green Approaches to Enhance Bioactive Compounds in Goji Berry (*Lycium barbarum*) Fruits: Comparative Optimization of Pressurized Water, Microwave‐Assisted, and Ultrasound‐Assisted Extraction Technologies by Using Response Surface Methodology

**DOI:** 10.1155/ijfo/8075181

**Published:** 2025-11-14

**Authors:** Ece Yildiz-Ozturk

**Affiliations:** ^1^ Department of Food Processing, Food Technology Programme, Yasar University, Bornova, Izmir, Turkey, yasar.edu.tr

**Keywords:** Goji berry, *Lycium barbarum*, polyphenolic compounds, green extraction, environment friendly, sustainability, optimization, response surface methodology (RSM)

## Abstract

Goji berries (*Lycium barbarum* L.), a superfruit with a long history of usage in Asian medicine, are gaining recognition for their potential as functional foods because of their high levels of antioxidants, flavonoids, anthocyanins, and phenolic acids. With the growing demand from consumers for clean‐label and naturally sourced ingredients, environmentally friendly extraction technologies are now crucial to creating bioactive‐rich extracts appropriate for food and nutraceutical applications. Three eco‐friendly extraction methods—pressurized water extraction (PWE), microwave‐assisted extraction (MAE), and ultrasound‐assisted extraction (UAE)—are thoroughly evaluated in this study to maximize the bioactive compounds’ recovery from Goji berry fruits. Water was the only solvent used in all extraction processes, guaranteeing environmental sustainability and food‐grade compliance. The solid/liquid ratio, temperature, duration, pressure, and power were all optimized using response surface methodology (RSM). The total phenolic content (TPC), total flavonoid content (TFC), total anthocyanin content (TAC), and antioxidant activity (DPPH inhibition) of the extracted materials were assessed. Under ideal circumstances, the extracts’ rutin contents were ascertained by HPLC analysis. According to the findings, MAE had the highest DPPH inhibition rate (75.942%), whereas PWE had the most TPC (17.753 mg GAE/g extract). The flavonoid content of both techniques was comparable. The UAE produced the best energy‐to‐bioactivity ratio and the most anthocyanin‐rich extracts (3.607 mg C3G/g). UAE is the most ecologically friendly option among the techniques, as evidenced by its highest overall efficiency in terms of bioactive recovery and antioxidant capacity. This is the first study to employ a combined approach of RSM and bioactivity‐energy efficiency assessment to optimize and compare water‐based PWE, MAE, and UAE methods for Goji berries. These results demonstrate that green extraction technologies can be leveraged to sustainably produce bioactive compounds from functional foods like Goji berries, which have significant applications in food, nutraceuticals, and cosmetics.

## 1. Introduction

Goji berries (*Lycium barbarum* L.) have been utilized in traditional Chinese medicine for generations, and they have gained more recognition in the Western world in the domains of nutraceuticals and functional foods in the last few years. Its fruits include high levels of organic acids, carotenoids, glucose, fructose, vitamin C, and phenolic compounds such as flavonoids and phenolic acids [1–3]. Numerous potential health benefits of this fruit have been found through research, including blood sugar regulation, eye health support, and antiaging effects. Goji berries’ health benefits include antitumor, immunomodulatory, anti‐inflammatory, and antioxidant qualities [4–7]. Because of these qualities, Goji berries are regarded as a “superfood” and are frequently used, particularly when dried, in food snacks, herbal teas, and functional food formulations [5, 7]. Goji berries are typically consumed in various forms, including fresh, dried, or freeze‐dried, and the majority of commercially available Goji products are in dried form. Traditional applications may involve washing, soaking or steeping, and thermal pretreatments [8–10]. While the traditional use of Goji berries is important, the extraction of bioactive compounds is critical for more effectively utilizing the potential of these compounds, increasing their concentrations, improving their bioavailability, and incorporating them into new‐generation functional foods or pharmaceutical products. The extraction of bioactive compounds enables them to be obtained in a more concentrated and standardized form [11]. Different solvents and extraction methods allow for the isolation of specific bioactive compounds or the same compounds in varying concentrations and with high purity. This facilitates the development of focused applications for specific health benefits [8]. Investigating the compounds’ potential for disease prevention and treatment requires the extraction process, which enables the isolation and additional study of compounds responsible for a variety of biological activities [2]. When exposed to heat, light, or oxygen, some bioactive substances—particularly unstable and lipophilic molecules like carotenoids—are more likely to degrade. Once these compounds have been extracted, their stability and bioavailability can be significantly increased by adding them to suitable delivery systems like nanoemulsions or nanocarriers [11]. When compared to dried Goji berries, extracts made using extraction techniques show a significant drop in the total microbial count, enhancing the products’ microbiological quality and consumer safety [8]. The pharmaceutical and functional food industries can use extracts to create new products because of their high concentrations of bioactive compounds and improved safety profiles [7, 12]. In this context, the aim of this study is to identify the most optimal sustainable extraction conditions for dried Goji berry extracts using green extraction techniques in terms of yield, biological activity, and energy consumption. This approach provides new data on the applicability of environmentally friendly extraction strategies and the potential for producing bioactive compound‐rich extracts at an industrial scale.

Sustainable extraction techniques are crucial for producing bioactive ingredients to lessen adverse effects on the economy and environment [13]. Traditional extraction methods’ use of organic solvents and high temperatures has specific detrimental consequences on the environment and public health. As a result, interest in green extraction techniques for environmental sustainability and human health has increased recently. Benefits of next‐generation extraction techniques include short processing times, low environmental costs, and extremely effective extraction when they only use environmentally friendly solvents like water [14]. Three distinct green extraction techniques are noteworthy in this regard. Effective extraction of bioactive compounds is made possible by pressurized water extraction (PWE), which uses water as a solvent under subcritical conditions in order to increase the diffusion rate. Most people concur that the method is affordable and environmentally beneficial [15, 16]. The microwave radiation utilized in microwave‐assisted extraction (MAE) speeds up the breaking of cell walls, allowing more bioactive compounds to enter the water. Food material’s water molecules are heated in microwaves, which speeds up and improves the release of bioactive ingredients. Shorter processing times and improved preservation of specific bioactive ingredients are the outcomes of this [17]. The sound waves used in ultrasound‐assisted extraction (UAE) cause bubbles in the liquid medium to suddenly implode, which encourages cell opening and the release of the bioactive substances inside. Higher antioxidant capacity is generally reported for the compounds obtained using this method [18]. With low energy consumption, MAE and UAE techniques in particular improve energy efficiency, minimize the use of organic solvents, and lower the carbon footprint on the environment. Sustainable green extraction techniques are also incorporated into recycling and waste management procedures, supporting sustainability objectives by providing important benefits like lowering the number of processing‐related byproducts and the environmental harm caused by these products. Green extraction methods ease the strain on the environment and use fewer resources. When analyzed from an economic standpoint, techniques created in accordance with sustainability principles also contribute to long‐term cost reduction. Additionally, it guarantees that natural resources are conserved without endangering the environment [19]. Thus, there is a lot of promise for both environmental and health benefits from the sustainable extraction of the bioactive ingredients of priceless superfoods like Goji berries. For scale‐up studies, the best outcomes are possible through comparison and optimization of extraction techniques.

There are several optimization studies in the literature for bioactive extraction from Goji berry [16, 18]. These studies have generally aimed to obtain high yields, phenolic compound content, and antioxidant activity. The yield obtained from hot water extraction (HWE) in the extraction of polysaccharides from Goji berries has been reported as 7.63%. The yield obtained using ultrasonic‐assisted subcritical water extraction (USWE) was reported to be 14.1%. This method provided a higher yield compared to HWE [20]. Additionally, USWE has been shown to provide higher polysaccharide yields compared to boiling water extraction, ultrasonic extraction in water, or subcritical water extraction [8]. The yield of MAE alone for *L. barbarum* leaf polysaccharides (LLP) was determined to be 0.891 ± 0.050*%*. It has been reported that LLP was obtained with a yield of 1.873 ± 0.001*%* under optimized conditions using ultrasonic–microwave combined extraction (UMCE). The UAE yield for LLP was reported as 1.182 ± 0.010*%* [15]. Experimental yields for the ultrasonic‐assisted enzymatic extraction (UAEE) of polysaccharides from Goji berries varied from 4.47% to 6.30%, with an ideal yield of 6.31% (±0.03%) under ideal circumstances [15]. Nevertheless, no research has been done in the literature that compares the yield, biological activity content, and energy efficiency of these three green extraction techniques (PWE, MAE, and UAE) when they are carried out with just water. The purpose of this work was to fill this significant gap by comparing the extracts obtained in terms of phenolic compounds, anthocyanins, and antioxidant activity, as well as to optimize PWE, MAE, and UAE techniques utilizing response surface methodology (RSM) to maximize the production of bioactive compounds from Goji berry fruits. RSM is an efficient tool that makes it possible to discover the ideal extraction conditions by systematically examining the effects of multiple experimental parameters (temperature, pressure, time, power, and solid/liquid ratio) [21]. The percentage yield of bioactive compounds and their beneficial health effects have been the main focus of future scale‐up studies and sustainable processing of Goji berries by identifying the ideal conditions. As a result, optimization research on the bioactive ingredients in Goji berries advances our understanding of this topic and benefits both industry and science. This research will provide a strong foundation for scaling up the extraction of Goji berries and their sustainable processing, supporting their potential in pharmaceutical and functional food applications.

## 2. Materials and Methods

### 2.1. Goji Berry Fruits

**Figure 1:**
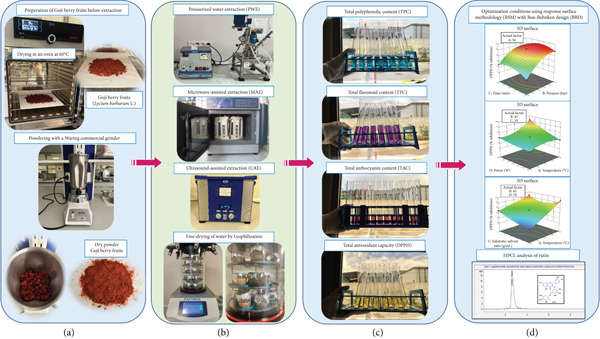
Schematic representation of green approaches to enhance bioactive compounds in Goji berry fruits. (a) Preparation of Goji berry fruits before extraction processes. (b) Pressurized water, microwave‐assisted, and ultrasound‐assisted extraction and lyophilization processes. (c) Biological activity analysis. (d) Optimization and modeling of extraction processes by using response surface methodology (RSM).

Goji berry fruits (*L. barbarum*) were supplied from the Goji berry Turkey production facility located in Korkuteli, Antalya. The fruits were dried for around 5 days at 60°C in an oven prior to the application of the extraction procedures. Next, a Waring laboratory‐scale commercial grinder was used to grind the dried Goji berry fruits into powder (Figure 1a). During the study, the dried fruits were stored in plastic ziplock bags at +4°C.

### 2.2. Materials and Reagents

Sigma provided the Folin–Ciocalteu’s reagent, 2,2‐diphenyl‐1‐picrylhydrazyl hydrate (DPPH), potassium acetate (CH_3_COOK), sodium carbonate (Na_2_CO_3_), and aluminum chloride (AlCl_3_). The in‐house nanopure water system was utilized to create the nanopure water used in the analysis. Quercetin and rutin, the standards used to identify flavonoids and phenolic acids, were acquired from Sigma‐Aldrich. Merck (Darmstadt, Germany) supplied the HPLC‐grade ethanol, acetonitrile, and methanol.

### 2.3. Procedures of Extraction

#### 2.3.1. PWE

High‐pressure HWE unit (Amar Equipment) and HPLC pump (Thermo Scientific, America) were used. Five grams of ground berry fruit and 50 mL of distilled water (1:10 [g/mL] ratio dried fruit powders/distilled water) were placed in the cartridge and placed in the 100‐mL extractor chamber. The mechanical shaft, which also includes the extractor and valves, was placed on top of each other and brought to the closed position. It was closed with clamps, and the screws were tightened. Before starting the extraction, the heater was turned on, and its temperature was increased to the desired temperature in the extraction trial. Since the extraction would be carried out in continuous flow, the desired flow rate (4 mL/min) was set in the HPLC pump, and when the desired temperature was reached, distilled water was pumped into the system to increase the pressure in the extractor. When the system reached high pressure (100‐150‐200 bar), the extraction process was started in a period of 30‐45‐60 min with the first flow in the sample outlet stream (Figure 1b). The RSM was used to build experimental sets based on three independent variables (temperature [40‐50‐60°C], pressure [100‐150‐200 bar], and extraction time [30‐45‐60 min]) in order to optimize the parameters for PWE.

#### 2.3.2. MAE

MAE was performed using a Microwave Digestion/Extraction Workstation system (Sineo Microwave Chemistry Technology Co. Ltd., China) with eight cartridges (50 mL chamber). Extraction time, temperature, power, and solid/liquid ratio parameters were all tuned to achieve high efficiency in MAE. In each working range, 2 g of dried berry fruits were combined with water in accordance with the solid/liquid ratio before being put into the cartridges (Figure 1b). The RSM methodology was used to build experimental sets based on four independent variables (temperature [40‐50‐60°C], extraction time [30‐45‐60 min], solid/liquid ratio [1:5‐1:10‐1:15 g/mL], and power [300‐600‐900 W]) in order to optimize the extraction parameters to be applied.

#### 2.3.3. UAE

UAE was carried out using the Elmasonic ultrasonic apparatus. Temperature, extraction time, power, and solid/liquid ratio parameters were all adjusted to achieve greater efficiency in ultrasonic‐assisted extraction. Using the solid‐to‐liquid ratio for each operating range, 2 g of dried berry fruits was combined with water and put into cartridges (Figure 1b). The RSM was used to build experimental sets based on four independent variables (temperature [40‐50‐60°C], extraction duration [30‐45‐60 min], solid/liquid ratio [1:5‐1:10‐1:15 g/mL], and power [40‐70‐100%]) in order to optimize the extraction conditions to be applied.

### 2.4. Biological Activity Analysis

The extracts obtained with three different extraction techniques were first frozen at −20°C, and then the water content of the extracts was removed using a lyophilizer device. All freeze‐dried extracts were weighed, and their amounts were determined, and then productivity calculations were made. The extracts were dissolved in distilled water and concentrated prior to biological activity assays (Figure 1b, c).

#### 2.4.1. Determination of Total Polyphenol Compounds (TPCs)

Gallic acid was used as a standard in the Folin–Ciocalteu reaction to determine the total quantities of polyphenols in the extracts. Prior to the extracts being analyzed, a calibration curve was created by creating sequential dilutions from the 1000 mg/L gallic acid main stock solution at concentrations of 500, 400, 300, 200, 100, 80, 60, 50, 40, 20, and 10 mg/L. Gallic acid (gallic acid equivalent [GAE]) per gram extract was used to express the results. The amount of GAE per gram of dry extract was used to express the total phenol concentration (standard curve equation : 760 nm = 0.0014.*c*_gallic acid_ [mg/mL] + 0.0208, *R* ^2^ = 0.999). Then, lyophilized Goji berry aqueous extracts were diluted fivefold and transferred to test tubes. First, 0.1 mL (100 *μ*L) berry extract and Folin–Ciocalteu reagent (500 *μ*L) were mixed in the test tubes. After mixing the resulting solution with a vortex, it was let to rest at room temperature for 5 min. Last but not least, distilled water was utilized to dilute the saturated Na_2_CO_3_ solution (1.5 mL, 20%) to 10 mL. After vortex mixing the test tubes, they were let to remain in the dark and at room temperature for an hour. The SP‐3000 nano‐OPTIMA spectrophotometer was used to test the reaction mixture’s absorbance at 760 nm.

#### 2.4.2. Determination of Total Flavonoid Content (TFC)

Flavonoids in the extracts react with the aluminum ion (Al^3+)^ to generate a stable flavonoid–Al^3+^ complex, which is the basis of the method’s premise. A yellow hue is produced by this complex, and the level of color intensity is correlated with the amount of flavonoids present. Sequential dilutions (MeOH) were made from 1000 mg/L quercetin main stock solution at concentrations of 700, 500, 400, 300, 200, 100, 80, 60, 50, 40, 20, and 10 mg/L, respectively, to produce a calibration curve prior to total flavonoid analysis of the extracts. The amount of flavonoids in an extract is measured in milligrams of quercetin equivalent per gram: (standard curve equation : 420 nm = 0.0078.*c*_quercetin_ [mg/mL] + 0.0012, *R* ^2^ = 0.999). The aqueous extracts of the Goji berries were then diluted five times and put into their tubes in order to measure the flavonoid concentration. The following ingredients were combined in test tubes and allowed to sit at room temperature for 30 min: 0.1 mL (100 *μ*L) of berry extract, followed by MeOH (3 mL), AlCl_3_ solution (0.2 mL, 10%), CH_3_COOK (0.2 mL, 1 M), and distilled water (5.6 mL). At 420 nm, the absorbance of the reaction mixture was measured with an SP‐3000 nano‐OPTIMA spectrophotometer.

#### 2.4.3. Determination of Total Anthocyanin Content (TAC)

A pH differential approach was used to determine the total quantity of anthocyanin. The foundation of this technique is the observation that anthocyanins exhibit varying pigmentations at varying pH levels. Anthocyanins are colorful and have the ability to transmit a variety of colors from orange to purple light at pH 1.0. All anthocyanins are colorless at pH 4.5. The anthocyanin molecule’s altered C ring is what causes this color shift. It is possible to measure these color variations based on pH using spectrophotometry. First, 0.0245 M potassium chloride was utilized, and HCl was used to bring the solution’s pH down to 1.00 in order to create a pH 1.00 buffer solution. 0.4 M sodium acetate was utilized, and HCl was employed to bring the solution’s pH down to 4.5 to create a pH 4.5 buffer solution. One milliliter of each extract was diluted five times for the anthocyanin analyses. To the samples, 4 mL of buffer solutions with pH values of 1.00 and 4.5 was added. The mixes of extract and buffer were then measured at 520 and 700 nm versus water. The content of anthocyanins is measured using the following formula and is given as milligram cyanidin‐3‐glucoside equivalents per gram dry weight of extract:

Anthocyanin pigment mgL=A×MW×DF×1000ε×l.

*Δ* *A* stands for absorbance difference, which is determined by the applicable method at pH 1.0 and pH 4.5. The following formula is used to determine the absorbance difference:

ΔA=A520700−Aat pH 1.0–A520700−A at pH 4.5.

Cyanidin‐3‐glucoside’s molecular weight (MW) is 449.2 g/mol, and its molar absorptivity (*ε*) is 26.900 L/mol.cm where L stands for layer thickness of absorbance measuring cuvette, centimeter (cuvette path length in centimeter) (10 mm = 1 cm), and DF for dilution factor.

#### 2.4.4. Determination of Total Antioxidant Capacity (DPPH Scavenging Activity)

This method’s foundation is the measurement of scavenging effects of compounds exhibiting antioxidant activity on the purple and stable DPPH radical. A 1 mM DPPH methanolic solution (0.5 mL) was added after the extracts had been diluted in 4 mL MeOH (20 *μ*L extract + 3980 *μ*L MeOH). After 15 s of stirring, the resultant liquid was left to sit at room temperature for 30 min in the dark. A SP‐3000 nano‐OPTIMA spectrophotometer was used to measure the reaction mixture’s absorbance against methanol at 517 nm following incubation. High free radical scavenging activity is shown by the reaction mixture’s low absorbance value. The sample concentration (IC_50_ value, grams per milliliter) needed to inhibit 50% of DPPH free radicals is determined by the percentage of DPPH radical scavenging inhibition. The absorbance readings are used to calculate the radical scavenging activity as a percentage that were read and entered into the provided equation:

%inhibition=ADPPH−AExtractADPPH×100

where *A*_DPPH_: absorbance of the control solution (methanolic DPPH solution) without extract and *A*_Ext_: absorbance of the extract‐containing solution.

### 2.5. Experimental Design and RSM

The interactions between independent and response variables are predicted and optimized using a statistical and mathematical method known as RSM in experimental operations. RSM is widely used, especially in the optimization of multivariate systems and process improvement [22].

One popular experimental design technique in RSM analysis is Box–Behnken design (BBD). BBD has basic advantages such as requiring fewer experiments, modeling without the need for experiments at extreme values, and being less costly, thus effective in practice [23, 24]. RSM and BBD are effective and efficient statistical approaches, especially for optimizing extraction processes. BBD serves as a powerful tool for modeling complex relationships between response variables and factors while lowering the quantity of tests.

The study investigated the effectiveness of various parameters—including temperature, pressure, time, solid/liquid ratio, and power—on the extraction of bioactive compounds from Goji berry fruit using three methods: PWE, MAE, and UAE. The optimization processes utilized a BBD and RSM to determine the ideal conditions that maximize biological activity values for each extraction method, as shown in Figure 1d. Three independent variables—temperature (40°C, 50°C, and 60°C), pressure (defined as 100‐150‐200 bar), and extraction time (30, 45, and 60 min)—were investigated in order to optimize PWE. Four independent variables were examined in the case of MAE: power (300‐600‐900 W), solid/liquid ratio (1:5, 1:10, and 1:15 g/mL), extraction time (30, 45, and 60 min), and temperature (40°C, 50°C, and 60°C). The same four independent variables were evaluated in a similar manner, with power for UAE being 40‐70‐100%. The RSM approach was used to build each trial set, and the Design‐Expert statistical tool (Design‐Expert 13.0.0) was used for optimization. Variance analysis (analysis of variance [ANOVA]), which included evaluating the inconsistency, fit coefficient (*R* ^2^), and Fisher test value (*F*‐value), was used to examine the precision of the model. The model is considered satisfactory if the regression rate is greater than 95%, which means that there is less than a 5% difference between the experimental and projected data. Furthermore, the importance of the chosen model terms is shown by a *p* value of less than 0.05. At a 95% confidence level, a factor is considered to have a significant effect on the process if its *p* value is less than 0.05 [16]. Even if the *R* ^2^ values are below 95%, if the model is statistically significant, this indicates that your model is not random and that the identified independent factors have a real effect on the responses [25]. In the study, the reliability of the model was evaluated not only using *R* ^2^ and adjusted *R* ^2^ values but also in conjunction with additional statistics such as the lack‐of‐fit and adequate precision. In the study, the lack‐of‐fit test was not significant (*p* > 0.05), and the adequate precision values were above 4. Based on these criteria, it was concluded that the models are satisfactory and have high predictive power, even if *R* ^2^ < 95*%*. Tables 1, 2, and 3 show the findings for the independent and experimental variables’ total polyphenol, flavonoid, anthocyanin content, antioxidant activity, and extraction efficiency.

**Table 1:** Box‐Behnken experimental design of extraction parameters for the optimization of biological activities of *Lycium barbarum* extracts obtained by PWE

**Exp. no**	**Temperature** **(** ^**o**^ **C)**	**Pressure** **(bar)**	**Time** **(min)**	**TPC** **(mg GAE/g)**	**TFC** **(mg QE/g)**	**TAC** **(mg C3G/g)**	**DPPH** **(% Inhibition)**	**Extraction Yield (%)**
1	50	200	60	5.71	0.849	1.606	46.30	23.92
2	40	200	45	7.6	0.888	2.004	43.33	26.67
3	50	200	30	17.75	1.137	1.992	48.02	31,28
4	60	150	30	7.8	0.902	1.562	31.91	27.22
5	50	150	45	8.2	1.032	1.226	52.10	35.34
6	60	150	60	7.49	0.933	1.762	34.51	18.96
7	50	100	60	10.72	0.824	1.088	24.75	13.05
8	40	150	60	8.46	0.872	1.312	36.79	21.01
9	60	100	45	7.87	0.838	1.118	37.72	22.77
10	40	150	30	8.6	0.778	1.342	35.74	24.40
11	60	200	45	7.48	0.926	1.902	38.89	24.42
12	50	100	30	9.1	0.785	1.448	17.53	10.07
13	40	100	45	10.96	0.833	1.392	13.15	7.65
14	50	150	45	9.42	1.067	1.135	49.63	34.69
15	50	150	45	9.19	1.042	1.280	50.59	29.64

Total polyphenolic content (TPC), Total flavonoid content (TFC), Total anthocyanin content (TAC), 2,2‐diphenyl‐1‐picrylhydrazyl (DPPH) radical scavenging activity

**Table 2:** Box‐Behnken experimental design of extraction parameters for the optimization of biological activities of *Lycium barbarum* extracts obtained by MAE

**Exp. no**	**Temperature** **(** ^**o**^ **C)**	**Time** **(min)**	**Substrate: Solvent** **(g/mL)**	**Power** **(W)**	**TPC** **(mg GAE/g)**	**TFC** **(mg QE/g)**	**TAC** **(mg C3G/g)**	**DPPH** **(% Inhibition)**	**Extraction Yield (%)**
1	40	45	5	600	5	0.652	0.069	32.18	30.19
2	40	45	15	600	9.38	0.662	0.136	41.91	45.94
3	50	30	10	900	10.6	0.560	0.772	40.51	51.35
4	50	60	10	900	9.67	0.612	0.120	43.61	52.06
5	60	60	10	600	8.71	0.747	0.458	49.45	63.86
6	60	45	10	900	8.67	0.851	0.169	84.61	61.77
7	50	45	5	900	9.73	0.547	0.301	40.02	41.58
8	50	30	10	300	9.36	0.614	0.410	40.75	45.86
9	50	45	15	900	10.43	0.521	0.162	41.30	51.61
10	50	45	10	600	10.09	0.640	0.455	42.40	50.43
11	50	60	15	600	10	0.441	0.391	36.80	53.40
12	40	45	10	900	8.42	0.785	1.110	42.27	43.25
13	50	45	10	600	10.04	0.635	0.609	42.15	54.83
14	60	30	10	600	10.97	0.597	0.239	47.81	61.17
15	50	45	10	600	10.28	0.662	0.556	44.59	48.84
16	50	60	5	600	10.44	0.533	0.567	28.89	33.16
17	50	30	15	600	9.76	0.601	0.079	45.38	52.65
18	40	60	10	600	10.53	0.504	0.776	34.67	45.74
19	50	30	5	600	10.11	0.512	0.252	13.20	16.54
20	60	45	15	600	10.14	0.487	0.220	32.73	56.97
21	60	45	5	600	12.1	0.882	0.977	44.40	44.86
22	60	45	10	300	9.4	0.489	0.645	34.00	61.49
23	50	45	5	300	9.49	0.574	0.052	26.46	40.27
24	50	45	15	300	8.26	0.620	1.042	40.69	62.09
25	50	60	10	300	7.94	0.525	0.215	39.84	58.19
26	40	45	10	300	7.5	0.604	0.631	28.47	33.11
27	40	30	10	600	6.94	0.600	0.119	28.16	34.94

Total polyphenolic content (TPC), Total flavonoid content (TFC), Total anthocyanin content (TAC), 2,2‐diphenyl‐1‐picrylhydrazyl (DPPH) radical scavenging activity

**Table 3:** Box‐Behnken experimental design of extraction parameters for the optimization of biological activities of *Lycium barbarum* extracts obtained by UAE

**Exp. no**	**Temperature** **(** ^**o**^ **C)**	**Time** **(min)**	**Substrate: Solvent** **(g/mL)**	**Power** **(%)**	**TPC** **(mg GAE/g)**	**TFC** **(mg QE/g)**	**TAC** **(mg C3G/g)**	**DPPH** **(% Inhibition)**	**Extraction Yield (%)**
1	50	30	10	40	7.41	0.689	0.503	52.73	53.92
2	50	45	5	40	9.31	0.672	0.137	37.68	30.46
3	50	60	15	70	7.09	0.838	0.707	55.51	64.98
4	40	45	15	70	8.64	0.760	1.342	35.30	57.56
5	60	45	5	70	9.24	0.754	1.992	54.49	40.88
6	50	30	10	100	10.41	0.808	1.395	52.83	56.87
7	50	30	5	70	14.41	0.784	1.024	34.39	28.55
8	60	60	10	70	7.06	0.905	2.138	48.84	64.44
9	60	45	15	70	4.96	0.767	0.449	53.64	92.96
10	40	60	10	70	5.92	0.634	1.277	46.82	50.67
11	50	45	15	40	10.92	0.744	1.118	51.01	55.99
12	40	30	10	70	6.97	0.722	0.749	30.15	52.98
13	60	45	10	40	6.17	0.775	0.197	52.07	63.65
14	50	60	10	100	6.12	0.885	0.290	51.67	64.68
15	40	45	10	100	6.92	0.643	1.024	28.89	50.94
16	60	30	10	70	7.92	0.825	1.031	47.02	60.74
17	40	45	10	40	8.82	0.750	2.631	26.46	49.99
18	50	45	10	70	6.76	0.848	1.265	33.23	56.09
19	50	30	10	70	7.48	0.759	0.618	43.33	60.75
20	60	45	10	100	8.12	0.879	0.143	43.03	58.29
21	50	60	5	70	7.60	0.788	1.049	44.90	43.79
22	50	45	10	70	7.82	0.833	1.090	36.46	59.38
23	50	45	10	70	7.82	0.833	1.031	32.73	62.79
24	50	45	15	100	9.60	0.822	1.143	49.49	63.89
25	50	60	10	40	8.44	0.673	0.780	34.44	58.89
26	50	45	5	100	8.17	0.916	0.686	43.48	51.76
27	40	45	5	70	7.48	0.717	0.914	17.93	34.27

Total polyphenolic content (TPC), Total flavonoid content (TFC), Total anthocyanin content (TAC), 2,2‐diphenyl‐1‐picrylhydrazyl (DPPH) radical scavenging activity

### 2.6. HPLC Analysis of *L. barbarum* Extracts Obtained Under Optimum Conditions

#### 2.6.1. Preparation of Samples

Five milliliters of water was used to dissolve 5 mg of subcritical, microwave, and ultrasonic water extracts that had been dried in a lyophilizer. The resulting mixture was then filtered through 0.45‐*μ*m nylon membrane filters (SRP 15, Machery, Germany). As a result, insoluble particles were eliminated and filtered.

#### 2.6.2. HPLC‐UV Conditions

The rutin content of the extracts prepared using different extraction methods was investigated in three replicates under HPLC‐UV conditions. HPLC‐UV analyses were conducted using an Agilent 1260 Infinity HPLC with an Agilent diode array detector (DAD) in Ege University Central Research Test and Analysis Laboratory Application and Research Center (EGE‐MATAL). A 4 × 150 mm Agilent Eclipse XDB C18.5 *μ*m column was utilized. The mobile phase consisted of methanol (v:v:v) (60:40:40) with 0.1% H_3_PO_4_ and water and acetonitrile. The column was allowed to acclimate to the initial conditions for 10 min before the following injection. The column temperature was set to ambient (30°C), and the detection wavelength, flow rate, and sample injection volume were set to 254 nm, 0.75 mL/min, and 20 *μ*L, respectively.

#### 2.6.3. Calibration

In the creation of the calibration graph, stock solutions of rutin standard were prepared. Five different concentrations of rutin were then obtained by diluting these stock solutions: 100, 500, 1000, 5000, and 10,000 ng/mL. Analysis was performed three times. A linear calibration graph was created by taking the average of the obtained values. A rutin calibration curve was created using peak area/concentration (ppb) to calculate the flavonoid amount of the relevant extract. The amount of rutin was expressed as nanograms per milliliter per extract. For rutin, standard curve equation : peak area = 0.0214.c_Rutin_ (ng/mL) − 3.7331, *R* ^2^ = 0.9994.

## 3. Results and Discussion

### 3.1. Modeling and Optimization of PWE

The PWE system is a technique that extracts active chemicals from biological and plant materials by using water as a solvent at high temperatures and pressures. This method offers an environmentally friendly, effective, and secure extraction procedure without the need for organic solvents. Working under 100–200 bar pressure and 40°C–60°C temperature conditions without switching to supercritical water prevents thermal degradation of sensitive ingredients. The stability of heat‐sensitive ingredients such as polyphenols, flavonoids, anthocyanins, and vitamins is especially preserved.

The high‐pressure water extraction system is influenced by several factors, including temperature, pressure, duration, solid/liquid ratio, and their interactions. Thus, determining the optimal extraction conditions requires statistical optimization. Total phenolics (milligrams of GAEs per gram extract), total flavonoids (milligrams of quercetin equivalents per gram extract), total anthocyanins (milligrams of cyanidin‐3‐glucoside per gram extract), and total antioxidant activity (% inhibition) results were expressed as functions of the independent variables using second‐order polynomial equations. Using ANOVA (*p* < 0.05) in combination with the tested and recommended extraction results, the best model was identified by contrasting a single factor’s two levels to assess the significant effects of these process variables. Statistical significance was established for each parameter, the regression coefficients of each biological activity response in the suggested experimental model were analyzed, and the model’s important and unimportant effects were assessed (Table 1).

In the extraction of bioactive compounds from Goji berries, temperature, pressure, and time each play different roles depending on the physicochemical properties and stability of the target compounds. These parameters must be carefully optimized and their interactions considered for optimal extraction efficiency and preservation of bioactive properties. The impact of temperature (40°C–60°C), pressure (100–200 bar), and extraction time (30–60 min) on the total polyphenol, flavonoid, anthocyanin content, and antioxidant activity of *L. barbarum* fruit extracts, as well as their interactions, was examined using a statistical experimental design (Figure 2). The least squares method was used to determine the regression coefficients for the experimental model’s linear, quadratic, intercept, and interaction components. The effect levels of each variable on the biological activity values were computed by establishing model equivalence for all factor levels. The coefficients of polynomial equivalencies of the first and second orders were used to compute and assess correlations between independent variables.

Figure 2Three‐dimensional (3D) response surface plots of PWE yield showing the effects of temperature, pressure, and time for (a) total polyphenolic content, (b) total flavonoid content, (c) total anthocyanin content, and (d) antioxidant activity.

**Figure:**
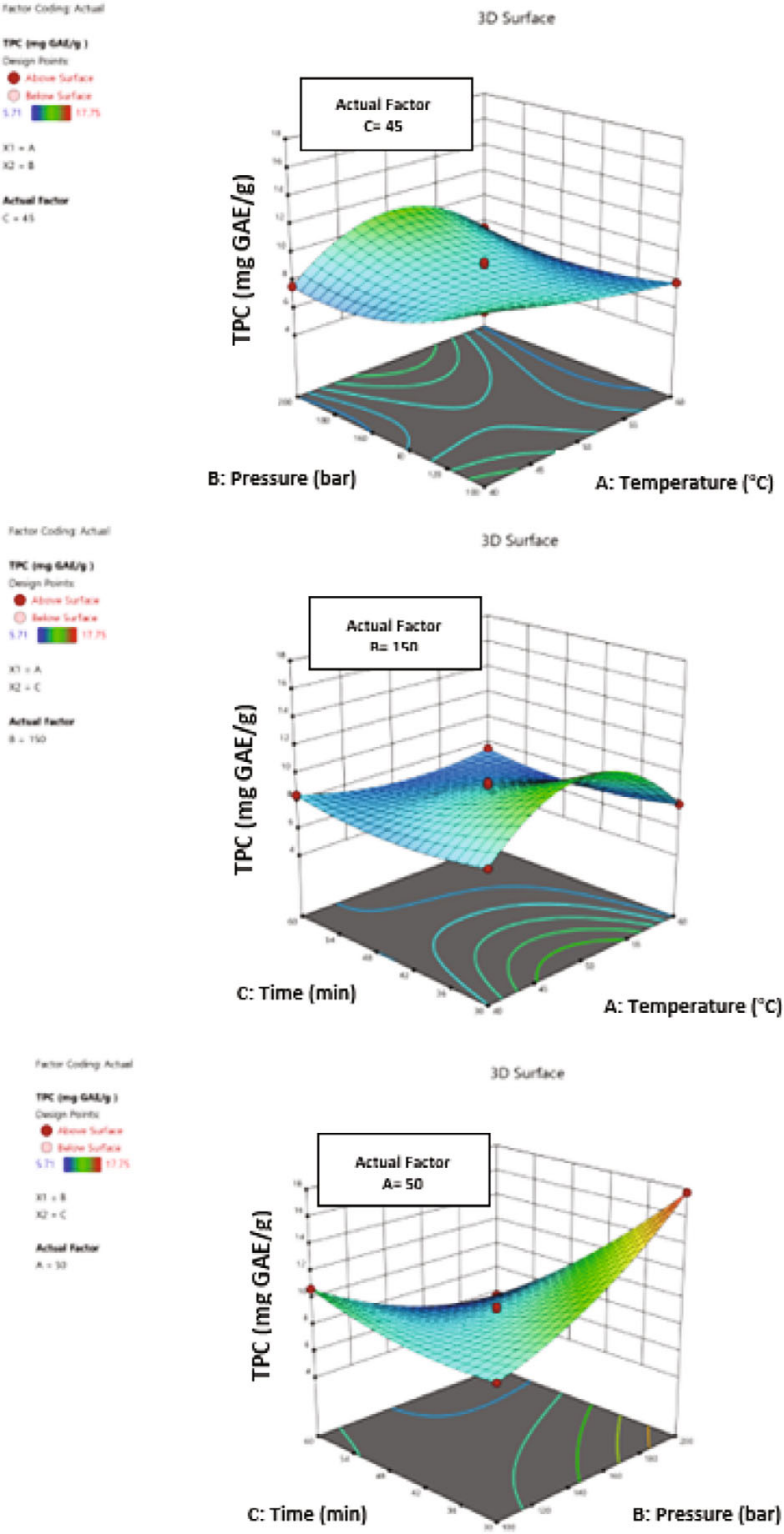
(a)

**Figure:**
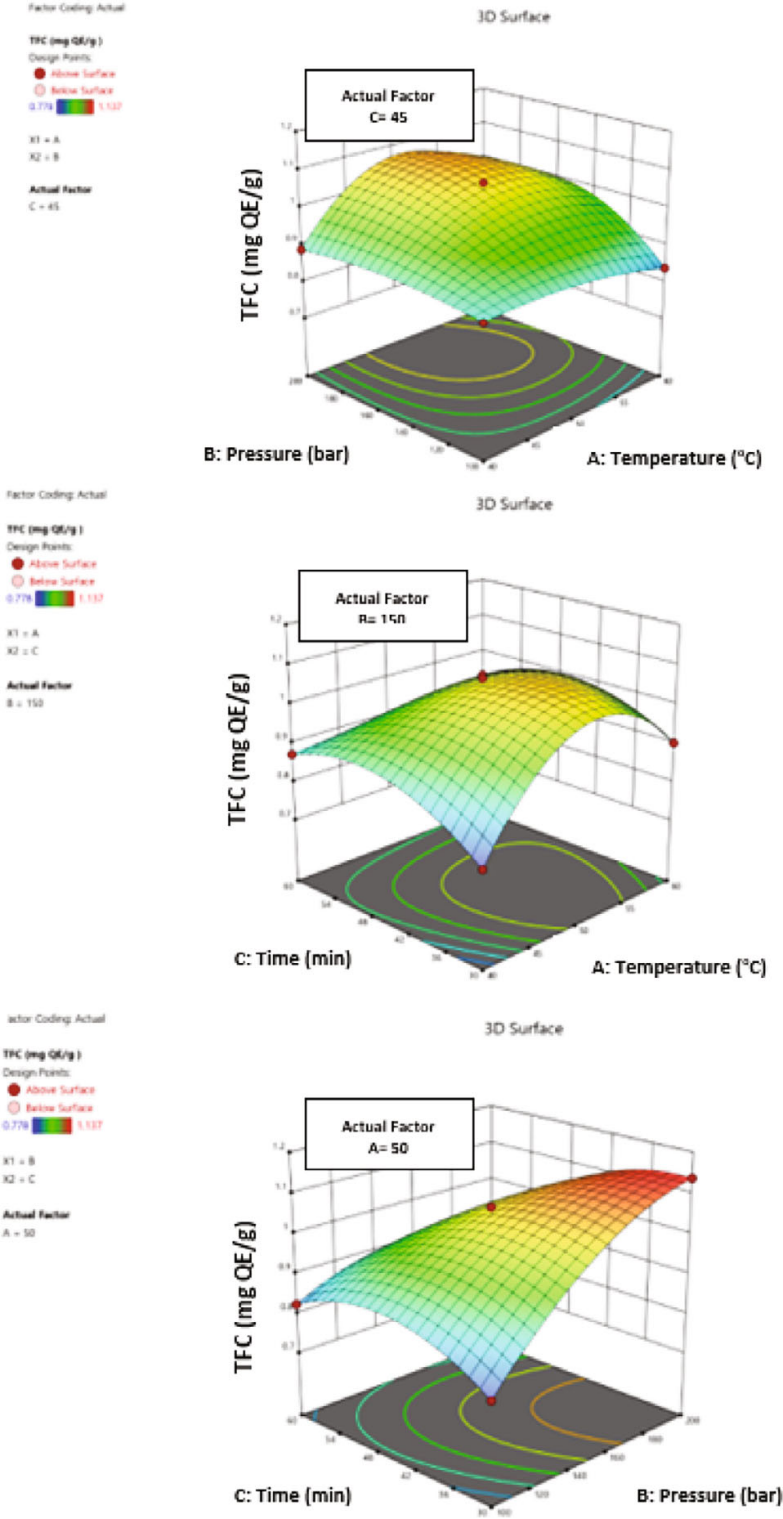
(b)

**Figure:**
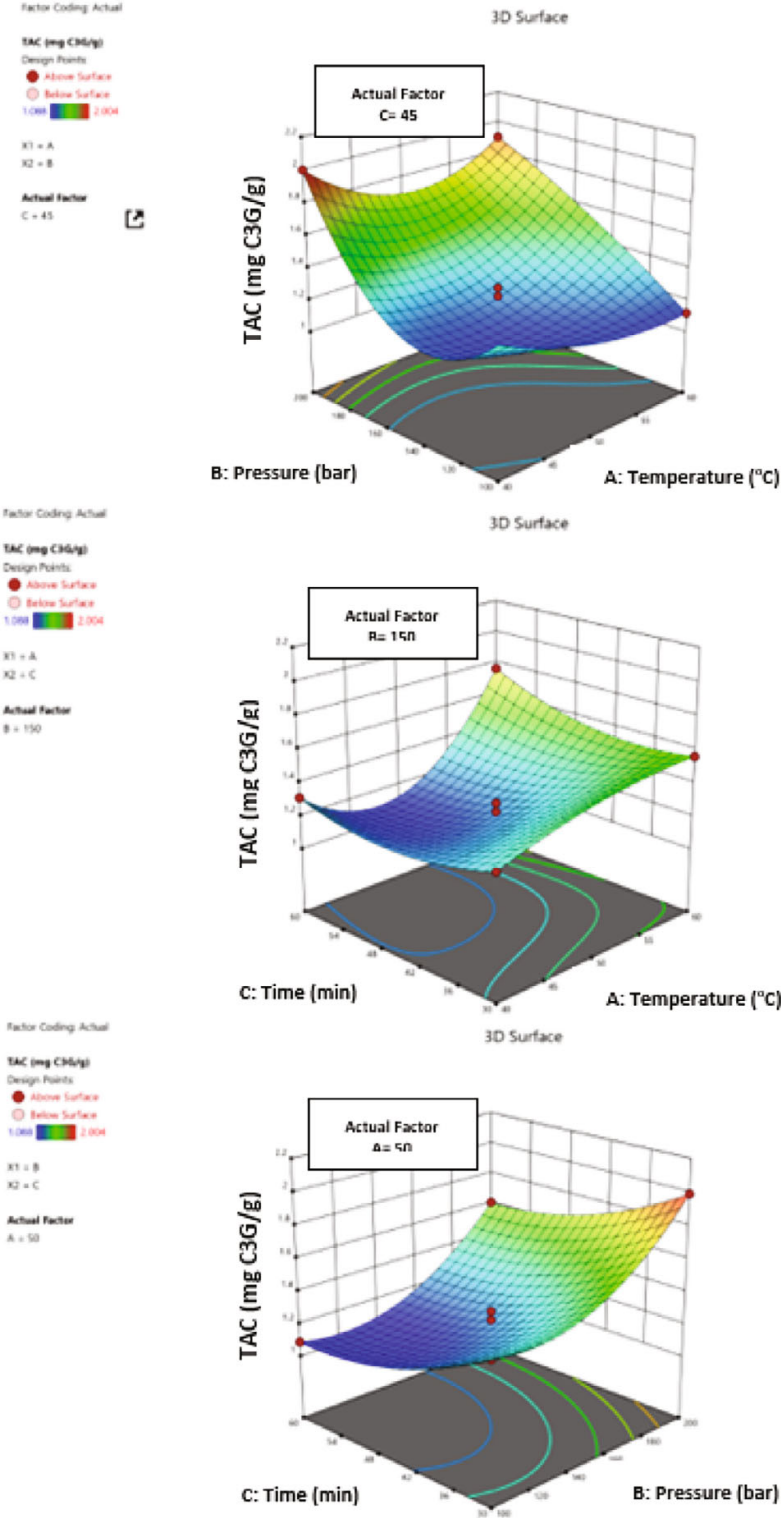
(c)

**Figure:**
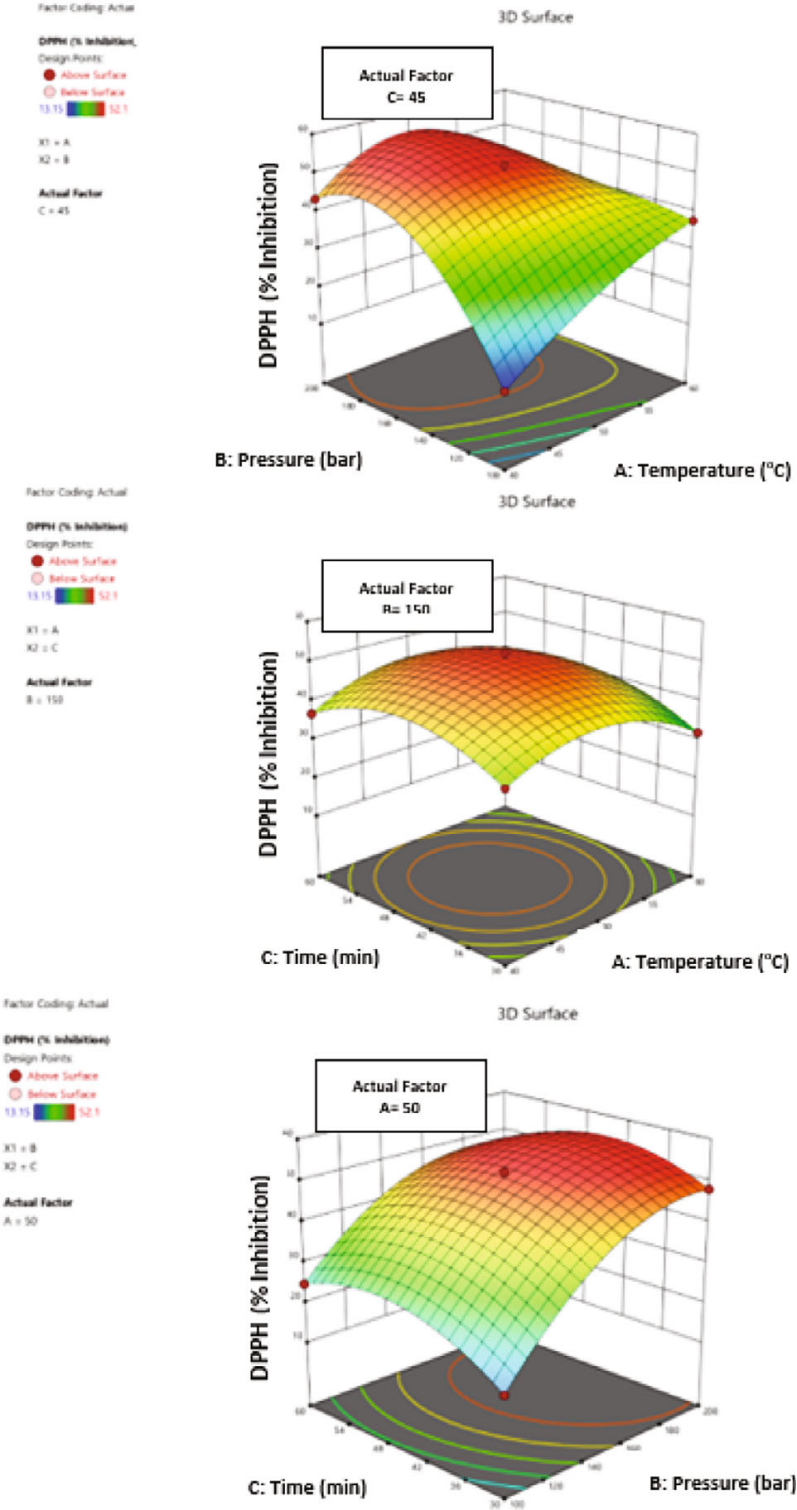
(d)

An ANOVA revealed that the applied part data for the cubic polynomial model was well represented considering available correlation coefficients at high resolution (*R* ^2^) for antioxidant activity, total polyphenolic, total flavonoid, and TAC being 0.9984, 0.9920, and 0.9960, respectively. Good agreement between the theoretical and experimental data derived from the suggested model is indicated by high *R* ^2^ values. To further show that the regression model fits the data well, *R* ^2^ and Adj − *R* ^2^ should be comparable. Table 2 shows that the *R* ^2^ and Adj − *R* ^2^ values in the models given for each response are close to each other. The model equations are sufficient to forecast yield when all of the variable values are combined, according to the statistical significance of the values of the four models created for biological activity (Y1, *p* : 0.0472^∗^ < 0.05; Y2, *p* : 0.0240^∗^ < 0.05; Y3, *p* : 0.0458^∗^ < 0.05; Y4, *p* : 0.0095^∗^ < 0.05).

The model terms are considered significant if the *p* value is less than 0.05 and not significant if the *p* value is more than 0.1. Important model terms in this case are C, BC, A^2^, and A^2^C. For the total polyphenol content alone, temperature (A) and pressure (B) were not statistically significant, but time (C) and pressure–time (*p* < 0.05) were. In terms of TFC, the following variables were statistically significant: temperature (A), pressure (B), time (C), and pressure–time (BC). Analysis of temperature (A), pressure (B), and time (C) in terms of the total amount of anthocyanin revealed that each of these factors was statistically significant on its own. When antioxidant activity was examined, it was discovered that the pressure (B) and temperature–pressure (AB) relationships were statistically significant (Table 4).

**Table 4:** Analysis of variance (ANOVA) for the fitted cubic polynomial model in pressurized water extraction (PWE).

**Source**	**Sum of Squares**	**df**	**Mean Square**	**F** **-value**	**p** **value**	**Source**	**Sum of Squares**	**df**	**Mean Square**	**F** **-value**	**p** **value**	**Source**	**Sum of Squares**	**df**	**Mean Square**	**F** **-value**	**p** **value**	**Source**	**Sum of Squares**	**df**	**Mean Square**	**F** **-value**	**p** **value**
**For Total Phenolic Content Model**	103.81	12	8.65	20.59	**0.0472**	**For Total Flavonoid Content Model**	0.1599	12	0.0133	41.01	**0.0240**	**For Total Anthocyanin Content Model**	1.37	12	0.1142	21.27	**0.0458**	**For DPPH % Inhibition Model**	1950.54	12	162.55	104.84	**0.0095**
A‐Temperature	0.7832	1	0.7832	1.86	0.3055	A‐Temperature	0.0086	1	0.0086	26.33	0.0359	A‐Temperature	0.1122	1	0.1122	20.90	0.0447	A‐Temperature	9.33	1	9.33	6.02	0.1336
B‐Pressure	3.31	1	3.31	7.88	0.1069	B‐Pressure	0.0355	1	0.0355	109.33	0.0090	B‐Pressure	0.2820	1	0.2820	52.50	0.0185	B‐Pressure	677.04	1	677.04	436.68	0.0023
C‐Time	27.14	1	27.14	64.59	0.0151	C‐Time	0.0155	1	0.0155	47.69	0.0203	C‐Time	0.1391	1	0.1391	25.91	0.0365	C‐Time	7.56	1	7.56	4.88	0.1579
AB	2.21	1	2.21	5.25	0.1491	AB	0.0017	1	0.0017	5 .30	0.1479	AB	0.0074	1	0.0074	1.38	0.3614	AB	210.40	1	210.40	135.70	0.0073
AC	0.0072	1	0.0072	0.0172	0.9077	AC	0.0010	1	0.0010	3.05	0.2227	AC	0.0132	1	0.0132	2.46	0.2571	AC	0.6006	1	0.6006	0.3874	0.5972
BC	46.65	1	46.65	111.01	0.0089	BC	0.0267	1	0.0267	82.25	0.0119	BC	0.0002	1	0.0002	0.0315	0.8755	BC	19.98	1	19.98	12.89	0.0696
A²	9.40	1	9.40	22.38	0.0419	A²	0.0336	1	0.0336	103.34	0.0095	A²	0.1139	1	0.1139	21.22	0.0440	A²	264.06	1	264.06	170.31	0.0058
B²	4.77	1	4.77	11.35	0.0779	B²	0.0170	1	0.0170	52.34	0.0186	B²	0.1701	1	0.1701	31.68	0.0301	B²	302.02	1	302.02	194.80	0.0051
C²	2.06	1	2.06	4.90	0.1573	C²	0.0239	1	0.0239	73.39	0.0134	C²	0.0408	1	0.0408	7.60	0.1102	C²	212.10	1	212.10	136.80	0.0072
ABC	0.0000	0				ABC	0.0000	0				ABC	0.0000	0				ABC	0.0000	0			
A²B	6.83	1	6.83	16.24	0.0564	A²B	0.0101	1	0.0101	31.02	0.0308	A²B	0.0139	1	0.0139	2.60	0.2484	A²B	53.51	1	53.51	34.51	0.0278
A²C	12.43	1	12.43	29.57	0.0322	A²C	0.0175	1	0.0175	53.80	0.0181	A²C	0.1049	1	0.1049	19.53	0.0476	A²C	0.4278	1	0.4278	0.2759	0.6518
AB²	0.2592	1	0.2592	0.6168	0.5145	AB²	0.0046	1	0.0046	14.18	0.0638	AB²	0.1368	1	0.1368	25.47	0.0371	AB²	86.07	1	86.07	55.51	0.0175
AC²	0.0000	0				AC²	0.0000	0				AC²	0.0000	0				AC²	0.0000	0			
B²C	0.0000	0				B²C	0.0000	0				B²C	0.0000	0				B²C	0.0000	0			
BC²	0.0000	0				BC²	0.0000	0				BC²	0.0000	0				BC²	0.0000	0			
A³	0.0000	0				A³	0.0000	0				A³	0.0000	0				A³	0.0000	0			
B³	0.0000	0				B³	0.0000	0				B³	0.0000	0				B³	0.0000	0			
C³	0.0000	0				C³	0.0000	0				C³	0.0000	0				C³	0.0000	0			
Pure Error	0.8405	2	0.4202			Pure Error	0.0006	2	0.0003			Pure Error	0.0107	2	0.0054			Pure Error	3.10	2	1.55		
Cor Total	104.65	14				Cor Total	0.1606	14				Cor Total	1.38	14				Cor Total	1953.64	14			
R²	0.9920					R²	0.9960					R²	0.9922					R²	0.9984				
Adjusted R²	0.9438					Adjusted R²	0.9717					Adjusted R²	0.9456					Adjusted R²	0.9889				
Adeq Precision	19.9506					Adeq Precision	21.3908					Adeq Precision	13.4267					Adeq Precision	32.4567				

The effectiveness of separate factors and their combined effects on polyphenolic compounds were examined by creating profiles of several nonlinear regression models’ three‐dimensional response surfaces. This allowed for the analysis of relationships between any two parameters and the efficient placement of the ideal parameter range, which maximized the total biological activities (Figure 2). The highest value of total polyphenolics and flavonoids was obtained at 50°C and 150 bar of pressure when the effects of temperature and pressure were studied for a set amount of time (45 min). At a fixed pressure of 150 bar, the effect of temperature and time was investigated; the highest value was obtained at 50°C for 45 min.

The highest value was obtained at 200 bar of pressure and 30 min when the effects of time and pressure were investigated at a fixed temperature (50°C) (Figure 2a,b). When the effects of temperature and pressure were investigated over a predetermined period of time (45 min), the highest value of total anthocyanins was obtained at a temperature of 40°C and a pressure of 200 bar. The best outcome was obtained at 40°C for 60 min when the effects of time and temperature were investigated at 150 bar of constant pressure. The highest result was achieved at 200 bar of pressure and 30 min when a constant temperature (50°C) was used to examine the effects of time and pressure (Figure 2c).

The highest level of antioxidant activity was attained at 40°C and 200 bar of pressure when the effects of temperature and pressure were studied for a predetermined amount of time (45 min). When the effects of temperature and time were investigated at constant pressure (150 bar), the highest result was obtained at 50°C for 45 min. The highest value was obtained at 150 bar of pressure and 45 min when the effects of pressure and time were investigated at a fixed temperature of 50°C (Figure 2d).

Regression analysis yielded the second‐degree polynomial equations that predict total polyphenols, flavonoids, anthocyanin contents, and antioxidant activity values. The temperature (°C), pressure (bar), and time (minute) are represented by A, B, and C, respectively, in the following equations:

Total polyphenolic content mg GAE/g extract=+8.940.44250.91002.610.74250.04253.421.601.140.74670.00001.852.490.36000.00000.00000.00000.00000.00000.0000−A+B−C+AB−AC−BC−A2+B2+C2+ABC−A2B+A2C−AB2+AC2+B2C+BC2+A3+B3+C3,Total flavonoid content mgQE/gextract=+1.050.04620.09430.06230.02080.01570.08170.09540.06790.08040.00000.07100.09350.04800.00000.00000.00000.00000.00000.0000+A+B−C+AB−AC−BC−A2−B2−C2+ABC−A2B+A2C−AB2+AC2+B2C+BC2+A3+B3+C3,Total anthocyanin content mgC3Ggextract=+1.210.16750.26550.18650.04300.05750.00650.17570.21470.10520.00000.08350.22900.26150.00000.00000.00000.00000.00000.0000+A+B−C+AB+AC−BC++B2+C2+ABC+A2B+A2C−AB2+AC2+B2C+BC2+A3+B3+C3,Antioxidant activity %Inhibition=+50.771.5313.011.377.250.38752.248.469.047.580.00005.170.46256.560.00000.00000.00000.00000.00000.0000−A+B+C−AB+AC−BC−A2−B2−C2+ABC−A2B−A2C+AB2+AC2+B2C+BC2+A3+B3+C3.

To validate the model’s predictive power, a final PWE was performed in ideal conditions in addition to the experiments. The Box–Behnken experimental design indicated that 49.5°C, 200 bar, and 30 min were the ideal extraction conditions for the highest biological activity values and extraction efficiency in PWE. These conditions resulted in 17.738 mg GAE/g, 1.135 mg QE/g, 1.997 mg C3G/g, 48.112% inhibition, and 30% extraction efficiency. 17.75 mg GAE/g was found to be the maximum total polyphenol content attained under the extraction experimental conditions of 50°C, 200 bar, and 30 min. Furthermore, 48.02% inhibition, 31.28% extraction efficiency, 1.137 mg QE/g, and 1.992 mg C3G/g were measured. Consequently, 50°C, 200 bar of pressure, and 30 min of extraction time were found to be the ideal extraction parameters for PWE. The findings supported the suitability of the established models and the experiments’ adherence to the ideal stage by demonstrating that the predicted and experimental values did not differ significantly (*p* > 0.05).

In the extraction of Goji berry (*L. barbarum*) fruit extracts, the most important model terms can generally be summarized as temperature, pressure, and extraction time, along with the interactions between these parameters. These terms are of critical importance due to their direct and interrelated effects on the yield and quality of bioactive compounds.

Temperature is one of the factors that most affects extraction efficiency [26]. As temperature increases, water viscosity decreases, and surface tension decreases. This allows water to penetrate the sample more effectively, increases the solubility of target bioactive compounds, and accelerates mass transfer, thereby facilitating the extraction of more compounds [13]. In HWE under pressure, as temperature increases, the dielectric constant (polarity) of water decreases significantly. This allows water to dissolve less polar compounds, which it normally tends to dissolve only polar compounds. This enables the extraction of medium‐polarity compounds such as phenolics and flavonoids [26]. The fact that the highest values of total polyphenols and flavonoids are obtained at 50°C indicates that this temperature is an ideal balance point for both increasing extraction and avoiding critical degradation [16]. These compounds may degrade at higher temperatures, reducing their antioxidant activity [25]. Anthocyanins, being heat‐sensitive compounds, may lose their color and structure through hydrolysis or pyrolysis at high temperatures. Therefore, anthocyanins exhibit their highest value at a lower temperature such as 40°C, where the risk of thermal degradation is lower [13]. Antioxidant activity is related to the total amount of bioactive compounds in the extract. The fact that the highest antioxidant activity is achieved at 40°C indicates that the protection of these sensitive compounds is critical for overall antioxidant capacity [12, 13].

Pressure is the primary factor affecting extraction kinetics and enabling water to remain in a liquid state at high temperatures [26]. It increases the capacity to extract less polar compounds by reducing the dielectric constant of water. High pressure can mechanically break down fruit cell walls, thereby accelerating the diffusion of intracellular bioactive compounds into water, increasing mass transfer, and consequently improving extraction efficiency [13, 21, 26]. The pressure of 150 bar, determined as optimal for total polyphenols and flavonoids, maximized yield by sufficiently disrupting cell structures and releasing phenolic compounds. However, when examined at a constant temperature (50°C), the highest value was obtained at 200 bar and 30 min, indicating that higher pressure application can increase efficiency by shortening the extraction time. The fact that the highest anthocyanin value was obtained at 200 bar pressure indicates that a stronger physical force (pressure) at a lower temperature is effective for the more efficient release of these sensitive compounds from the cells. The fact that 200 bar pressure yields the highest value for antioxidant activity indicates that this pressure optimizes the extraction of both sensitive anthocyanins and other phenolics.

A sufficient extraction time is also necessary for the target bioactive compounds to completely dissolve in water and diffuse from the matrix. However, after a certain period, equilibrium is reached between the matrix and the solvent, and further, time does not significantly increase extraction efficiency [26]. Very long extraction times, especially under intense conditions such as high temperature or pressure, can increase the risk of degradation of bioactive compounds due to thermal degradation, oxidation, or enzyme activity [25]. A 45‐min duration, determined as optimal for total polyphenols and flavonoids, has been shown to achieve maximum extraction without significant degradation. However, the fact that a 30‐min duration at 200 bar pressure and 50°C yields the highest yield suggests that a shorter duration at higher pressure may be sufficient and even preferable to prevent degradation. For anthocyanins, a 60‐min duration at 40°C and 150 bar constant pressure yielding the best results indicates that a longer duration at lower temperature and moderate pressure helps maximize the extraction of these heat‐sensitive compounds while preventing degradation [13]. The 45‐min duration at 50°C and 150 bar pressure yielding the highest antioxidant activity indicates that the overall antioxidant compound profile is best preserved and extracted within this timeframe.

### 3.2. Modeling and Optimization of MAE

MAE is a contemporary extraction technology that makes it possible to quickly, effectively, and sustainably extract bioactive substances—particularly phenolic compounds and anthocyanins—from plant and fruit sources. Microwaves generate heat by exciting the water molecules in the solvent and plant cell. This heat breaks down the cell walls and releases the contents. Microwave rays (300 MHz–300 GHz) cause rotation and friction of water and other polar molecules. This friction results in rapid heat generation. As intracellular pressure rises, the cell wall disintegrates. The solvent absorbs the bioactive substances therein.

A multitude of variables, including microwave power, the solid/liquid ratio, time, temperature, solvent, and the interplay among these variables, influence the MAE system. The best extraction conditions must therefore be found by statistical optimization. Total phenolics (milligrams of GAEs per gram extract), total flavonoids (milligrams of quercetin equivalents per gram extract), total anthocyanins (milligrams of cyanidin‐3‐glucoside per gram extract), and total antioxidant activity (% inhibition) were all plotted against independent variables using second‐degree polynomial equations. Two levels of a single component were examined using ANOVA (*p* < 0.05) and assessed, and recommended extraction findings were used to identify the best model and the significant effects of process variables. The statistical significance of each parameter was ascertained, the regression coefficients of the suggested experimental model were analyzed for every biological activity response, and the model’s significant and insignificant impacts were assessed (Table 5).

**Table 5:** Analysis of variance (ANOVA) for the fitted cubic polynomial model in microwave‐assisted extraction (MAE).

**Source**	**Sum of Squares**	**df**	**Mean Square**	**F** **-value**	**p** **value**	**Source**	**Sum of Squares**	**df**	**Mean Square**	**F** **-value**	**p** **value**	**Source**	**Sum of Squares**	**df**	**Mean Square**	**F** **-value**	**p** **value**	**Source**	**Sum of Squares**	**df**	**Mean Square**	**F** **-value**	**p** **value**
**For Total Phenolic Content Model**	52.34	22	2.38	107.93	**0.0002**	**For Total Flavonoid Content Model**	0.2931	22	0.0133	9.58	**0.0202**	**For Total Anthocyanin Content Model**	2.45	22	0.1114	6.34	**0.0426**	**For DPPH** **% Inhibition Model**	3718.56	22	169.03	94.47	**0.0002**
**A-Temperature**	1.16	1	1.16	52.42	0.0019	**A-Temperature**	0.0006	1	0.0006	0.4317	0.5470	**A-Temperature**	0.2148	1	0.2148	12.24	0.0249	**A-Temperature**	572.88	1	572.88	320.21	< 0.0001
**B-Time**	1.38	1	1.38	62.63	0.0014	**B-Time**	0.0003	1	0.0003	0.2461	0.6458	**B-Time**	0.1794	1	0.1794	10.22	0.0330	**B-Time**	1.20	1	1.20	0.6702	0.4590
**C-Substrate:Solvent ratio**	0.0702	1	0.0702	3.19	0.1488	**C-Substrate:Solvent ratio**	0.0001	1	0.0001	0.0719	0.8018	**C-Substrate:Solvent ratio**	0.1811	1	0.1811	10.31	0.0325	**C-Substrate:Solvent ratio**	60.14	1	60.14	33.61	0.0044
**D-Power**	1.45	1	1.45	65.87	0.0013	**D-Power**	0.0040	1	0.0040	2.85	0.1664	**D-Power**	0.0995	1	0.0995	5.67	0.0759	**D-Power**	50.20	1	50.20	28.06	0.0061
AB	8.56	1	8.56	388.12	< 0.0001	AB	0.0151	1	0.0151	10.88	0.0300	AB	0.0480	1	0.0480	2.73	0.1737	AB	5.93	1	5.93	3.31	0.1428
AC	10.05	1	10.05	455.86	< 0.0001	AC	0.0410	1	0.0410	29.49	0.0056	AC	0.1697	1	0.1697	9.67	0.0359	AC	114.49	1	114.49	63.99	0.0013
AD	0.6806	1	0.6806	30.88	0.0051	AD	0.0082	1	0.0082	5.89	0.0722	AD	0.2280	1	0.2280	12.99	0.0227	AD	338.74	1	338.74	189.34	0.0002
BC	0.0020	1	0.0020	0.0919	0.7769	BC	0.0082	1	0.0082	5.89	0.0722	BC	2.250E‐06	1	2.250E‐06	0.0001	0.9915	BC	147.26	1	147.26	82.31	0.0008
BD	0.0600	1	0.0600	2.72	0.1743	BD	0.0050	1	0.0050	3.57	0.1316	BD	0.0522	1	0.0522	2.97	0.1597	BD	4.02	1	4.02	2.25	0.2083
CD	0.9312	1	0.9312	42.24	0.0029	CD	0.0013	1	0.0013	0.9321	0.3890	CD	0.3187	1	0.3187	18.15	0.0130	CD	41.93	1	41.93	23.43	0.0084
A²	4.44	1	4.44	201.64	0.0001	A²	0.0159	1	0.0159	11.46	0.0276	A²	0.0006	1	0.0006	0.0343	0.8621	A²	20.72	1	20.72	11.58	0.0272
B²	0.0001	1	0.0001	0.0042	0.9514	B²	0.0277	1	0.0277	19.93	0.0111	B²	0.0947	1	0.0947	5.39	0.0809	B²	106.92	1	106.92	59.76	0.0015
C²	0.0042	1	0.0042	0.1886	0.6865	C²	0.0127	1	0.0127	9.15	0.0390	C²	0.1232	1	0.1232	7.02	0.0570	C²	309.37	1	309.37	172.92	0.0002
D²	2.61	1	2.61	118.27	0.0004	D²	0.0012	1	0.0012	0.8727	0.4031	D²	0.0023	1	0.0023	0.1292	0.7374	D²	25.93	1	25.93	14.49	0.0190
ABC	0.0000	0				ABC	0.0000	0				ABC	0.0000	0				ABC	0.0000	0			
ABD	0.0000	0				ABD	0.0000	0				ABD	0.0000	0				ABD	0.0000	0			
ACD	0.0000	0				ACD	0.0000	0				ACD	0.0000	0				ACD	0.0000	0			
BCD	0.0000	0				BCD	0.0000	0				BCD	0.0000	0				BCD	0.0000	0			
A²B	1.69	1	1.69	76.79	0.0009	A²B	0.0010	1	0.0010	0.7445	0.4369	A²B	0.3711	1	0.3711	21.14	0.0100	A²B	4.44	1	4.44	2.48	0.1903
A²C	1.09	1	1.09	49.35	0.0022	A²C	0.0205	1	0.0205	14.75	0.0185	A²C	0.2968	1	0.2968	16.91	0.0147	A²C	38.06	1	38.06	21.27	0.0099
A²D	0.6161	1	0.6161	27.95	0.0061	A²D	0.0559	1	0.0559	40.24	0.0032	A²D	0.0502	1	0.0502	2.86	0.1660	A²D	315.51	1	315.51	176.35	0.0002
AB²	0.0005	1	0.0005	0.0204	0.8933	AB²	0.0104	1	0.0104	7.51	0.0519	AB²	0.0664	1	0.0664	3.78	0.1236	AB²	22.58	1	22.58	12.62	0.0237
AC²	4.08	1	4.08	184.88	0.0002	AC²	0.0014	1	0.0014	0.9724	0.3799	AC²	0.4603	1	0.4603	26.22	0.0069	AC²	251.22	1	251.22	140.41	0.0003
AD²	0.0000	0				AD²	0.0000	0				AD²	0.0000	0				AD²	0.0000	0			
B²C	0.0084	1	0.0084	0.3833	0.5693	B²C	0.0001	1	0.0001	0.0476	0.8380	B²C	0.1800	1	0.1800	10.25	0.0328	B²C	75.52	1	75.52	42.21	0.0029
B²D	0.0392	1	0.0392	1.78	0.2532	B²D	0.0032	1	0.0032	2.27	0.2061	B²D	0.1008	1	0.1008	5.74	0.0747	B²D	14.15	1	14.15	7.91	0.0482
BC²	1.07	1	1.07	48.35	0.0022	BC²	0.0013	1	0.0013	0.9353	0.3882	BC²	0.2716	1	0.2716	15.47	0.0171	BC²	3.03	1	3.03	1.69	0.2633
BD²	0.0000	0				BD²	0.0000	0				BD²	0.0000	0				BD²	0.0000	0			
C²D	0.0000	0				C²D	0.0000	0				C²D	0.0000	0				C²D	0.0000	0			
CD²	0.0000	0				CD²	0.0000	0				CD²	0.0000	0				CD²	0.0000	0			
Residual	0.0882	4	0.0220			Residual	0.0056	4	0.0014			Residual	0.0702	4	0.0176			Residual	7.16	4	1.79		
**Lack of Fit**	0.0561	2	0.0281	1.75	0.3637	Lack of Fit	0.0051	2	0.0026	12.48	0.0742	Lack of Fit	0.0580	2	0.0290	4.74	0.1743	Lack of Fit	3.55	2	1.78	0.9857	0.5036
Pure Error	0.0321	2	0.0160			Pure Error	0.0004	2	0.0002			Pure Error	0.0122	2	0.0061			Pure Error	3.60	2	1.80		
Cor Total	52.43	26				Cor Total	0.2987	26				Cor Total	2.52	26				Cor Total	3725.72	26			
R²	0.9983					R²	0.9814					R²	0.9721					R²	0.9981				
Adjusted R²	0.9891					Adjusted R²	0.8790					Adjusted R²	0.8189					Adjusted R²	0.9875				
Adeq Precision	51.8124					Adeq Precision	12.1675					Adeq Precision	8.5851					Adeq Precision	57.8441				

The effects of temperature (40°C–60°C), extraction time (30–60 min), solid/liquid ratio (1/5–1/15), and microwave power (300–900 W) on the total polyphenol, flavonoid, anthocyanin content, and antioxidant activity of *L. barbarum* fruit extracts and their interactions were characterized using a statistical experimental design (Figure 3). ANOVA showed that the applied data from the cubic polynomial model component were well represented, with high‐resolution correlation coefficients (*R* ^2^) of 0.9983, 0.9814, 0.9721, and 0.9981 for total polyphenolic, total flavonoid, total anthocyanin, and antioxidant activity, respectively. Excellent coordination between the theoretical and findings from the trial derived from the suggested model is indicated by high *R* ^2^ values. Additionally, a good fit of the regression model should be indicated by comparable *R* ^2^ and Adj − *R* ^2^. The *R* ^2^ and Adj − *R* ^2^ values for each response in the provided models are reasonably close to one another, as indicated in Table 4. The statistically significant values of the four models created for biological activity (Y1, *p* : 0.0002^∗^ < 0.05; Y2, *p* : 0.0202^∗^ < 0.05; Y3, *p* : 0.0426^∗^ < 0.05; Y4, *p* : 0.0002^∗^ < 0.05) indicate that the model equations are sufficient to predict the yield under any set of variable values.

Figure 3Three‐dimensional (3D) response surface plots of MAE yield showing the effects of temperature, time, solid/liquid ratio and microwave power for total polyphenolic content (a), for total flavonoid content (b), for total anthocyanin content (c) and for antioxidant activity (d).

**Figure:**
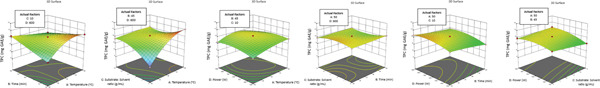
(a)

**Figure:**
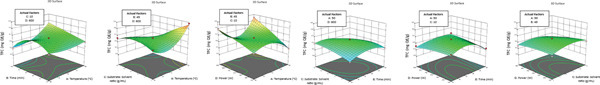
(b)

**Figure:**
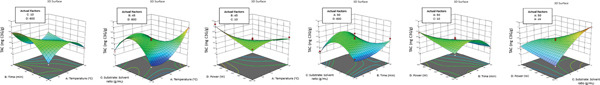
(c)

**Figure:**
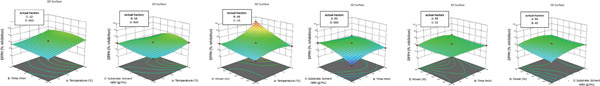
(d)

If the *p* value is less than 0.05, the model terms are deemed significant; if it is larger than 0.1, they are deemed not significant. Key model terms for total polyphenol content are A, B, D, AB, AC, AD, and CD. However, temperature (A), time (B), power (D), temperature–time (AB), temperature–solid/liquid ratio (AC), temperature–power (AD), and solid/liquid ratio–power (CD) were all found to be significant (*p* < 0.05), although solid/liquid ratio (C) (*p* > 0.05) was not significant in terms of statistics on its own (Table 4). While temperature (A), time (B), solid/liquid ratio (C), and power (D) were not significant on their own, temperature–time (AB) and temperature–solid/liquid ratio (AC) interactions were statistically significant when measured in terms of TFC. When measured in terms of the total amount of anthocyanin, the solid/liquid ratio (C), temperature (A), and time (B) were all determined to be statistically significant on their own. Additionally, significant were the correlations between temperature and power (AD), solid/liquid ratio and power (CD), and temperature and solid/liquid ratio (AC). Antioxidant activity analysis revealed that temperature (A), power (D), and the solid/liquid ratio (C) were all statistically significant on their own. Important interactions included the solid/liquid ratio–power (CD), time–solid/liquid ratio (BC), temperature–solid/liquid ratio (AC), and temperature–power (AD) (Table 5).

When power (D: 600 W) and constant solid/liquid ratio (C: 1/10) were examined, the maximum amount of total polyphenolics (10.97 mg/g, *local maximum for this factor combination*) (Figure 3a) was achieved after 30 min at 60°C. The consequences of continuous time (B: 45 min) and power (D: 600 W) were evaluated, and the maximum value (12.1 mg/g) was found at 60°C and a solid/liquid ratio of 1: 5. With constant duration (B: 45 min) and solid/liquid ratio (C: 1/10) examined, the highest value (10.28 mg/g, *local maximum for this factor combination*) was reached at 50°C and 600 W of power. Following an investigation into the effects of constant temperature (A: 50°C) and power (D: 600 W), the highest value (10.44 mg/g, *local maximum for this factor combination*) was obtained at 60 min and a solid/liquid ratio of 1:5. When the effects of constant temperature (A: 50°C) and solid/liquid ratio (C: 1/10) were examined, the maximum value (10.6 mg/g, *local maximum for this factor combination*) was found at 30 min and 900 W of power. When the effects of time (B: 45 min) and steady temperature (A: 50°C) were investigated, the maximum value (10.43 mg/g) was found at 1/15 solid/liquid ratio and 900 W power. These values represent partial maxima from two‐factor interaction surfaces and should not be interpreted as the global optimum.

The maximum result (0.747 mg/g) of total flavonoids (Figure 3b) was obtained at 60°C and 60 min after controlling for the consequences of power (D: 600 W) and constant solid/liquid ratio (C: 1/10). Following an investigation into the effects of constant time (B: 45 min) and power (D: 600 W), the highest value (0.882 mg/g) was found at 60°C and a solid/liquid ratio of 1: 5. Investigating the effects of time (B: 45 min) and solid/liquid ratio (C: 1/10) resulted in the highest value (0.851 mg/g) at 60°C and 900 W power. After 45 min and a solid/liquid ratio of 1:10, the effects of constant temperature (A: 50°C) and power (D: 600 W) were examined, and the maximum value (0.662 mg/g) was attained. Following an investigation into the effects of constant temperature (A: 50°C) and solid/liquid ratio (C: 1/10), the greatest value (0.662 mg/g) was reached at 45 min and 600 W of electricity. With constant temperature (A: 50°C) and time (B: 45 min), the highest value (0.662 mg/g) was obtained at 1/10 solid/liquid ratio and 600 W power.

When power (D: 600 W) and a constant solid/liquid ratio (C: 1/10) were considered, the maximum amount of total anthocyanin (0.776 mg/g) (Figure 3c) was achieved at 40°C and 60 min. Upon analyzing the impact of continuous time (B: 45 min) and power (D: 600 W), the highest value (0.977 mg/g) was obtained at 60°C and a 1:5 solid/liquid ratio. The greatest value (1.11 mg/g) was obtained at 40°C and 900 W of power when the impacts of the solid/liquid ratio (C: 1/10) and continuous time (B: 45 min) were examined. Forty‐five minutes later, with a 1:10 solid/liquid ratio, the effects of constant temperature (A: 50°C) and power (D: 600 W) were examined, and the highest value (0.609 mg/g) was attained. Following an analysis of the effects of a constant temperature (A: 50°C) and the solid/liquid ratio (C: 1/10), the maximum value (0.772 mg/g) was found at 30 min and 900 W of power. After examining the effects of a constant temperature (A: 50°C) and duration (B: 45 min), the greatest value (1.042 mg/g) was reached at 1/15 solid/liquid ratio and 300 W power.

The maximum value (49.45%) of antioxidant activity (Figure 3d) was obtained at 60°C and 60 min when the impacts of a constant solid/liquid ratio (C: 1/10) and power (D: 600 W) were considered. The temperature of 50°C and the solid‐to‐liquid ratio of 1:10 yielded the highest value (44.59%) when the effects of constant time (B: 45 min) and power (D: 600 W) were examined. Investigating the effects of continuous time (B: 45 min) and solid/liquid ratio (C: 1/10) yielded the highest value (84.61%) at 60°C and 900 W of electricity. Investigating the effects of continuous temperature (A: 50°C) and power (D: 600 W) yielded the highest value (45.38%) at 30 min and a solid/liquid ratio of 1:15. The highest value (44.59%) was obtained at 45 min and 600 W of power when the effects of solid/liquid ratio (C: 1/10) and constant temperature (A: 50°C) were examined. With constant temperature (A: 50°C) and time (B: 45 min), a solid‐to‐liquid ratio of 1/10 and a power of 600 W produced the highest value (44.59%).

The regression analysis below shows the second‐degree polynomial equations that were used to estimate the amounts of total polyphenol, total flavonoid, total anthocyanin, and antioxidant activity. In the equations, the letters A, B, C, and D stand for temperature (°C), time (minute), solid/liquid ratio (grams per milliliter), and power (watt).

Total polyphenolic content mg GAE/g extract=+10.140.53750.58750.13250.60251.461.580.41250.02250.12250.48250.91290.00420.02790.69920.00000.00000.00000.00000.92000.73750.55500.01501.430.00000.06500.14000.73000.00000.00000.00000.00000.00000.00000.0000+A−B−C+D−AB−AC−AD−BC+BD+CD−A2−B2−C2−D2+ABC+ABD+ACD+BCD+A2B+A2C−A2D+AB2+AC2+AD2−B2C+B2D+BC2+BD2+C2D+CD2+A3+B3+C3+D3,Total flavonoid content mg QE/g extract=+0.64570.01220.00930.00500.03150.06150.10130.04520.04520.03520.01800.05470.07210.04880.01510.00000.00000.00000.00000.02280.10130.16720.07230.02600.00000.00580.03970.02550.00000.00000.00000.00000.00000.00000.0000−A−B+C−D+AB−AC+AD−BC+BD−CD+A2−B2−C2−D2+ABC+ABD+ACD+BCD+A2B−A2C+A2D+AB2+AC2+AD2−B2C+B2D−BC2+BD2+C2D+CD2+A3+B3+C3+D3,Total anthocyanin content mg C3G/g extract=+0.54000.23170.21180.21270.15770.10950.20600.23870.00080.11430.28220.01060.13330.15200.02060.00000.00000.00000.00000.43080.38520.15850.18230.47970.00000.30000.22450.36850.00000.00000.00000.00000.00000.00000.0000−A−B+C−D−AB−AC−AD−BC−BD−CD+A2−B2−C2+D2+ABC+ABD+ACD+BCD+A2B−A2C+A2D+AB2+AC2+AD2−B2C+B2D+BC2+BD2+C2D+CD2+A3+B3+C3+D3,Antioxidant activity %inhibition=+43.0511.970.54753.883.541.225.359.206.071.003.241.974.487.622.200.00000.00000.00000.00001.494.3612.563.3611.210.00006.152.661.230.00000.00000.00000.00000.00000.00000.0000+A+B+C+D−AB−AC+AD−BC+BD−CD+A2−B2−C2+D2+ABC+ABD+ACD+BCD+A2B−A2C+A2D−AB2−AC2+AD2+B2C−B2D+BC2+BD2+C2D+CD2+A3+B3+C3+D3.

To confirm the model’s predictive ability, a final MAE was conducted under ideal circumstances in addition to the experiments. The optimal extraction parameters for the maximum biological activity levels and extraction efficiency in MAE were 60°C, 48.276 min, 1:5 g/mL solid/liquid ratio, and 899.996 W power, based on the experimental design of Box–Behnken. These conditions resulted in 10.549 mg GAE/g, 1.077 mg QE/g, 1.197 mg C3G/g, 75.942% inhibition, and 48.930% extraction efficiency. Thus, under the extraction experimental operating conditions, the closest parameters were 60°C, 45 min, 1:10 g/mL solid/liquid ratio, and 900 W power. 84.61% inhibition was achieved with 8.67 mg GAE/g, 0.851 mg QE/g, and 0.169 mg C3G/g. As a result, the ideal extraction parameters for MAE were identified as 60°C, 45 min, 1/5 g/mL solid/liquid ratio, and 900 W of power. The findings demonstrated that the experimental and predicted values did not differ significantly (*p* > 0.05), confirming that the constructed models were appropriate and that the experiments followed the best stage.

The bioactive compounds and biological activity values obtained from the extraction of Goji berries vary significantly depending on many factors, such as the type of Goji used, extraction method, solvent, and optimized parameters. Parameters such as temperature, microwave power, duration, and solid/liquid ratio in the extraction process have significant effects on the quantity and quality of the bioactive compounds obtained. Each of these parameters must be carefully controlled to obtain the best yield as they have varying effects on the extraction mechanism. The TFC and particularly the TAC values are lower than those reported in some black Goji berry studies [27, 28], but overall, the TPC and antioxidant activity values obtained are comparable to or consistent with those reported in some studies in the literature [13, 29]. This could be the result of particular optimization differences in extraction techniques or inherent phytochemical differences between black Goji (*Lycium ruthenicum*) and red Goji (*L. barbarum*), such as the high anthocyanin content in black Goji. Particularly, anthocyanins are important bioactive substances that make up a large portion of the polyphenol content of black Goji berries [11, 12, 27].

Temperature is a crucial factor that directly affects mass transfer and the solubility of compounds in extraction processes. In general, raising the temperature speeds up the release of target compounds from cells and improves the solvent’s ability to penetrate plant material. This makes it possible to extract more bioactive substances, like polyphenols [25]. Excessively high temperatures can sometimes make extraction less effective. Temperatures above 60°C, for instance, have been shown to decrease enzyme activity in polysaccharide extraction, which lowers yield [30]. Heat‐sensitive phenolic compounds, like anthocyanins, can degrade at high temperatures [31]. Anthocyanins have been observed to degrade at temperatures higher than 50°C. The cytotoxic effect and antioxidant activity of the extract can be diminished by prolonged or unreasonably high temperature exposure, which can change the chemical structure of bioactive compounds (like antioxidants) [13, 25]. Although the study’s moderate‐to‐high temperature of 60°C produced positive results for polyphenolic compounds and antioxidant activity, there may be a chance that anthocyanins will degrade. This effect is in line with the study’s findings of an increase in flavonoid (0.882 mg/g) and total polyphenol (12.1 mg/g) levels at 60°C. However, research indicates that anthocyanin degradation kinetics speed up at high temperatures, indicating that the ideal temperature range is between 40°C and 60°C.

Utilizing the quick and uniform heating effect brought on by microwave radiation, MAE operates. One crucial factor that has a direct impact on the effectiveness of extraction and the release of bioactive compounds is microwave power. Through dipole rotation and ionic conductivity, the microwave field rapidly heats cells; it also speeds up mass transfer by causing microexplosions and increasing the permeability of cell walls and membranes. This improves extraction efficiency by facilitating the release of intracellular bioactive compounds into the solvent [6, 32]. Power can boost efficiency up to a point, but too much power can degrade sensitive compounds (like anthocyanins and flavonoids) and cause local overheating. Thus, it is crucial to balance temperature, time, and microwave power [33–35]. The study found that at 40°C and 900 W of power, the anthocyanin content rose to 1.11 mg/g. This result is in line with earlier research showing that compound release can be accelerated even in brief periods of time by high microwave power. However, local overheating brought on by excessive power can reduce biological activity.

The amount of time needed for the solvent to permeate the plant matrix, dissolve the target compounds, and diffuse is determined by the extraction time. More bioactive compounds can typically be transferred into the extract by lengthening the extraction time. However, overly lengthy extraction times can cause thermally unstable compounds to degrade, especially increasing losses from oxidation/isomerization in some flavonoids and anthocyanins. Furthermore, extraction yield reaches saturation after a certain point, and additional time extension has no positive effects on efficiency or the economy [25]. Polyphenols and flavonoids in the study reached nearly maximum levels with an extraction time of 30–45 min, whereas in some cases, a 60‐min duration resulted in a decrease in compound content.

The ratio of the amount of solvent to the amount of plant material is known as the solid/liquid ratio. The concentrations of the extracted material, extraction efficiency, and process cost are all significantly impacted by this ratio. Higher extraction efficiency and better mass transfer are typically provided by larger solvent volumes (lower solid/liquid ratios). This can be attributed to the solvent’s increased ability to extract target compounds from the matrix [15, 25]. This may, however, result in a drop in the extracted extract’s concentration and raise the price of further purification procedures. But when the solid/liquid ratio is raised too much, the extract becomes diluted, which reduces the driving force, and back diffusion/equilibrium may cause efficiency to drop [36]. The polyphenol yield in the study increased significantly to 12.1 mg/g at a solid/liquid ratio of 1:5 g/mL.

In terms of the polyphenol and antioxidant activity values obtained, microwave extraction parameters—particularly temperature and power—are comparable to those found in related studies in the literature. However, because Goji berries vary in type and may degrade with temperature, anthocyanin levels may be lower than in some studies. Careful optimization is crucial in these kinds of studies because of the intricate relationships between temperature and microwave power and the stability and extraction of bioactive compounds [25]. In actual extraction processes, these parameters interact with one another even though each one has a separate impact on the extraction mechanism. For instance, a higher temperature brought on by microwave power may shorten the time or raise the possibility of compound degradation. In order to optimize extraction yield and quality, statistical optimization techniques are commonly employed because of these intricate interactions [37].

### 3.3. Modeling and Optimization of UAE

UAE is carried out by subjecting a liquid system to high‐frequency sound waves, typically between 20 and 100 kHz. The high‐frequency sound waves emitted from the ultrasonic device create cavitation bubbles in the liquid. When these bubbles grow and burst, they create micro jets, shock waves, and local high‐temperature/pressure areas. The cell walls are mechanically broken down by this process, allowing the bioactive substances within to enter the solvent.

The frequency of ultrasonic waves, temperature, extraction time, power, solvent, solid/liquid ratio, and the interactions between these parameters all have an impact on the UAE system. Therefore, the optimal extraction conditions must be determined by statistical optimization. TPC (mg GAE/g extract), TFC (mg QE/g extract), TAC (mg C3G/g extract), and total antioxidant activity (% inhibition) were all expressed as functions of the independent variables using second‐degree polynomial equations. By comparing two levels of a single factor, testing, and suggested extraction results using the ANOVA (*p* < 0.05), the best model and the significant effects of process variables were identified. The statistical significance of each parameter was ascertained, the regression coefficients for every biological activity response were analyzed, and the model’s significant and insignificant impacts were assessed (Table 6).

**Table 6:** Analysis of variance (ANOVA) for the fitted cubic polynomial model in ultrasound‐assisted extraction (UAE).

**Source**	**Sum of Squares**	**df**	**Mean Square**	**F** **-value**	**p** **value**	**Source**	**Sum of Squares**	**df**	**Mean Square**	**F** **-value**	**p** **value**	**Source**	**Sum of Squares**	**df**	**Mean Square**	**F** **-value**	**p** **value**	**Source**	**Sum of Squares**	**df**	**Mean Square**	**F** **-value**	**p** **value**
**For Total Phenolic Content Model**	87.68	22	3.99	8.72	**0.0240**	**For Total Flavonoid Content Model**	0.1531	22	0.0070	10.17	**0.0181**	**For Total Anthocyanin Content Model**	8.95	22	0.4069	20.06	**0.0050**	**For DPPH** **% Inhibition Model**	2590.66	22	117.76	7.70	**0.0301**
**A-Temperature**	0.5256	1	0.5256	1.15	0.3439	**A-Temperature**	0.0170	1	0.0170	24.88	0.0076	**A-Temperature**	2.75	1	2.75	135.47	0.0003	**A-Temperature**	395.02	1	395.02	25.83	0.0071
**B-Time**	2.66	1	2.66	5.82	0.0734	**B-Time**	0.0009	1	0.0009	1.36	0.3085	**B-Time**	0.1714	1	0.1714	8.45	0.0438	**B-Time**	94.58	1	94.58	6.18	0.0677
**C-Substrate:Solvent ratio**	2.31	1	2.31	5.06	0.0878	**C-Substrate:Solvent ratio**	0.0001	1	0.0001	0.1768	0.6958	**C-Substrate:Solvent ratio**	0.5170	1	0.5170	25.49	0.0072	**C-Substrate:Solvent ratio**	93.51	1	93.51	6.11	0.0687
**D-Power**	1.51	1	1.51	3.31	0.1429	**D-Power**	0.0259	1	0.0259	37.87	0.0035	**D-Power**	0.0824	1	0.0824	4.06	0.1141	**D-Power**	4.58	1	4.58	0.2995	0.6133
AB	0.0090	1	0.0090	0.0198	0.8950	AB	0.0071	1	0.0071	10.31	0.0326	AB	0.0838	1	0.0838	4.13	0.1118	AB	55.13	1	55.13	3.61	0.1304
AC	7.40	1	7.40	16.19	0.0158	AC	0.0002	1	0.0002	0.3287	0.5971	AC	0.9712	1	0.9712	47.89	0.0023	AC	82.99	1	82.99	5.43	0.0803
AD	3.71	1	3.71	8.11	0.0465	AD	0.0111	1	0.0111	16.26	0.0157	AD	0.6030	1	0.6030	29.73	0.0055	AD	32.89	1	32.89	2.15	0.2164
BC	10.30	1	10.30	22.55	0.0090	BC	0.0014	1	0.0014	2.05	0.2251	BC	0.0010	1	0.0010	0.0505	0.8332	BC	0.6972	1	0.6972	0.0456	0.8414
BD	7.08	1	7.08	15.49	0.0170	BD	0.0022	1	0.0022	3.16	0.1502	BD	0.4775	1	0.4775	23.54	0.0083	BD	73.36	1	73.36	4.80	0.0937
CD	0.0081	1	0.0081	0.0177	0.9005	CD	0.0069	1	0.0069	10.06	0.0338	CD	0.0686	1	0.0686	3.38	0.1396	CD	13.40	1	13.40	0.8760	0.4023
A²	3.75	1	3.75	8.20	0.0457	A²	0.0127	1	0.0127	18.58	0.0125	A²	0.2116	1	0.2116	10.43	0.0320	A²	0.6135	1	0.6135	0.0401	0.8510
B²	0.3019	1	0.3019	0.6607	0.4619	B²	0.0037	1	0.0037	5.45	0.0798	B²	0.0447	1	0.0447	2.21	0.2117	B²	307.07	1	307.07	20.08	0.0110
C²	8.29	1	8.29	18.14	0.0131	C²	0.0034	1	0.0034	4.90	0.0912	C²	0.1035	1	0.1035	5.11	0.0867	C²	128.21	1	128.21	8.38	0.0443
D²	2.51	1	2.51	5.48	0.0792	D²	0.0059	1	0.0059	8.59	0.0428	D²	0.4206	1	0.4206	20.74	0.0104	D²	145.98	1	145.98	9.55	0.0366
ABC	0.0000	0				ABC	0.0000	0				ABC	0.0000	0				ABC	0.0000	0			
ABD	0.0000	0				ABD	0.0000	0				ABD	0.0000	0				ABD	0.0000	0			
ACD	0.0000	0				ACD	0.0000	0				ACD	0.0000	0				ACD	0.0000	0			
BCD	0.0000	0				BCD	0.0000	0				BCD	0.0000	0				BCD	0.0000	0			
A²B	0.2278	1	0.2278	0.4986	0.5191	A²B	0.0006	1	0.0006	0.8694	0.4039	A²B	0.7583	1	0.7583	37.39	0.0036	A²B	179.93	1	179.93	11.77	0.0265
A²C	4.74	1	4.74	10.38	0.0322	A²C	0.0008	1	0.0008	1.11	0.3513	A²C	0.8147	1	0.8147	40.17	0.0032	A²C	0.9940	1	0.9940	0.0650	0.8113
A²D	0.7875	1	0.7875	1.72	0.2595	A²D	0.0132	1	0.0132	19.29	0.0118	A²D	0.6244	1	0.6244	30.79	0.0052	A²D	14.82	1	14.82	0.9694	0.3806
AB²	1.57	1	1.57	3.43	0.1377	AB²	0.0016	1	0.0016	2.33	0.2015	AB²	2.48	1	2.48	122.50	0.0004	AB²	54.39	1	54.39	3.56	0.1324
AC²	0.0276	1	0.0276	0.0604	0.8179	AC²	0.0059	1	0.0059	8.60	0.0427	AC²	1.53	1	1.53	75.51	0.0010	AC²	28.69	1	28.69	1.88	0.2426
AD²	0.0000	0				AD²	0.0000	0				AD²	0.0000	0				AD²	0.0000	0			
B²C	13.73	1	13.73	30.05	0.0054	B²C	0.0003	1	0.0003	0.4034	0.5599	B²C	0.5973	1	0.5973	29.45	0.0056	B²C	0.0055	1	0.0055	0.0004	0.9858
B²D	1.23	1	1.23	2.70	0.1759	B²D	0.0000	1	0.0000	0.0148	0.9091	B²D	0.0037	1	0.0037	0.1823	0.6913	B²D	21.29	1	21.29	1.39	0.3034
BC²	1.94	1	1.94	4.25	0.1084	BC²	0.0001	1	0.0001	0.0884	0.7810	BC²	0.1109	1	0.1109	5.47	0.0795	BC²	221.97	1	221.97	14.52	0.0189
BD²	0.0000	0				BD²	0.0000	0				BD²	0.0000	0				BD²	0.0000	0			
C²D	0.0000	0				C²D	0.0000	0				C²D	0.0000	0				C²D	0.0000	0			
CD²	0.0000	0				CD²	0.0000	0				CD²	0.0000	0				CD²	0.0000	0			
Residual	1.83	4	0.4569			Residual	0.0027	4	0.0007			Residual	0.0811	4	0.0203			Residual	61.17	4	15.29		
Lack of Fit	1.08	2	0.5393	1.44	0.4099	Lack of Fit	0.0026	2	0.0013	17.25	0.0548	Lack of Fit	0.0515	2	0.0257	1.74	0.3651	Lack of Fit	52.97	2	26.48	6.46	0.1340
Pure Error	0.7491	2	0.3745			Pure Error	0.0002	2	0.0001			Pure Error	0.0296	2	0.0148			Pure Error	8.20	2	4.10		
Cor Total	89.51	26				Cor Total	0.1559	26				Cor Total	9.03	26				Cor Total	2651.82	26			
R²	0.9796					R²	0.9824					R²	0.9910					R²	0.9769				
Adjusted R²	0.8673					Adjusted R²	0.9875					Adjusted R²	0.9416					Adjusted R²	0.8501				
Adeq Precision	14.3641					Adeq Precision	11.6781					Adeq Precision	19.8176					Adeq Precision	11.2590				

The effects of temperature (40°C–60°C), extraction time (30–60 min), solid/liquid ratio (1/5–1/15), and ultrasonic power (40%–100%) on the antioxidant activity, flavonoid, anthocyanin, and total polyphenol content of Goji berry fruit extracts, as well as their interactions, were characterized using a statistical experimental design (Figure 4). The correlation coefficients (*R* ^2^) for total polyphenolic, total flavonoid, total anthocyanin, and antioxidant activity were found to be 0.9796, 0.9824, 0.9910, and 0.9769, respectively. An ANOVA analysis confirmed that the data derived from the cubic polynomial model component were accurately represented. A high *R* ^2^ score indicates that the theoretical and experimental results from the suggested model accord well. Additionally, a regression model that fits well should be indicated by comparable *R* ^2^ and Adj − *R* ^2^. *R* ^2^ and Adj − *R* ^2^ values are near to one another in the models provided for each response, as indicated in Table 6. The statistically significant values of the four models created for biological activity (Y1, *p* : 0.0240^∗^ < 0.05; Y2, *p* : 0.0181^∗^ < 0.05; Y3, *p* : 0.0050^∗^ < 0.05; Y4, *p* : 0.0301^∗^ < 0.05) indicate that the model equations are sufficient to predict the yield under any set of variable values.

Figure 4Three‐dimensional (3D) response surface plots of UAE yield showing the effects of temperature, time, solid/liquid ratio and ultrasound power for total polyphenolic content (a), for total flavonoid content (b), for total anthocyanin content (c) and for antioxidant activity (d).

**Figure:**
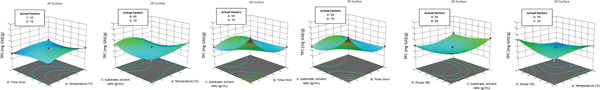
(a)

**Figure:**
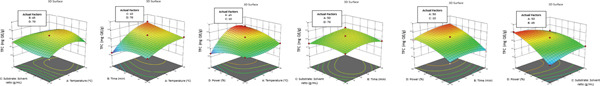
(b)

**Figure:**
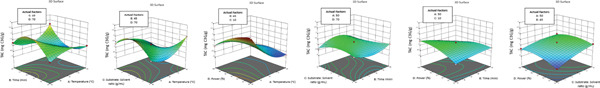
(c)

**Figure:**
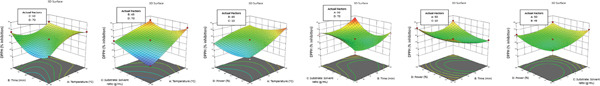
(d)

The model terms are considered significant if the *p* value is less than 0.05; if it is greater than 0.1, they are deemed not significant. For total polyphenol content, the temperature–solid/liquid ratio (AC), temperature–power (AD), time–solid/liquid ratio (BC), and time–power (BD) interactions were significant (*p* < 0.05), but temperature (A), time (B), solid/liquid ratio (C), and power (D) were not statistically significant on their own (Table 6). For total flavonoid content, while time (B) and solid/liquid ratio (C) by themselves were not statistically significant, temperature (A), power (D), temperature–time (AB), temperature–power (AD), and solid/liquid ratio–power (CD) interactions were statistically significant. Although power (D) alone was not significant, the temperature (A), time (B), and power (D) parameters, along with the temperature–time (AB), temperature–solid/liquid ratio (AC), temperature–power (AD), and time–power (BD) interactions, were found to be statistically significant when examined in terms of total anthocyanin content. Temperature (A) alone was statistically significant in terms of antioxidant activity, whereas time (B), solid/liquid ratio (C), and power (D) were not (Table 6).

The highest value (7.92 mg/g, *local maximum for this factor combination*) for total polyphenolics (Figure 4a) was obtained at 60°C for 30 min after the impacts of power (D: 70%) and constant solid/liquid ratio (C: 1/10) were examined. The highest value (9.24 mg/g, *local maximum for this factor combination*) was discovered at 60°C and a 1:5 solid/liquid ratio following an investigation into the effects of time (B: 45 min) and power (D: 70%). Finding the maximum value (8.82 mg/g, *local maximum for this factor combination*) at 40% power and 40°C was achieved by examining the effects of continuous time (B: 45 min) and solid/liquid ratio (C: 1/10). Investigating the effects of constant temperature (A: 50°C) and power (D: 70%) yielded the highest value (14.41 mg/g) at 30 min and a 1:5 solid/liquid ratio. When the impacts of constant temperature (A: 50°C) and solid/liquid ratio (C: 1/10) were examined, the highest value (10.41 mg/g, *local maximum for this factor combination*) was obtained at 30 min and 100% power, respectively. Examining the effects of constant temperature (A: 50°C) and time (B: 45 min), the highest value (10.92 mg/g) was found at 40% power and 1/15 solid/liquid ratio. These values represent partial maxima from two‐factor interaction surfaces and should not be interpreted as the global optimum.

The highest value (0.905 mg/g) for total flavonoids (Figure 4b) was obtained at 60°C for 60 min after analyzing the effects of a constant solid/liquid ratio (C: 1/10) and power (D: 70%). At 50°C and a 1:10 solid/liquid ratio, the highest value (0.848 mg/g) was obtained after examining the effects of constant power (D: 70%) and time (B: 45 min). Fixed time (B: 45 min) and solid/liquid ratio (C: 1/10) effects were examined, and the highest value (0.879 mg/g) was reached at 60°C and 100% power. The highest value (0.848 mg/g) was discovered at 45 min and a solid/liquid ratio of 1/10 when the effects of fixed temperature (A: 50°C) and power (D: 70%) were investigated. The highest value (0.885 mg/g) was attained at 60 min and 100% power when the effects of fixed temperature (A: 50°C) and solid/liquid ratio (C: 1/10) were investigated. The highest value (0.916 mg/g) was obtained at 100% power and a 1/5 solid/liquid ratio after examining the effects of set time (B: 45 min) and temperature (A: 50°C).

The maximum value (2.138 mg/g) of total anthocyanin quantity (Figure 4c) was obtained at 60°C for 60 min when the effects of a constant solid/liquid ratio (C: 1/10) and power (D: 70%) were considered. Upon analyzing the impact of continuous time (B: 45 min) and power (D: 70%), the highest value (1.992 mg/g) was obtained at 60°C and a 1/5 solid/liquid ratio. The effects of continuous time (B: 45 min) and solid/liquid ratio (C: 1/10) were examined, and the maximum value (2.631 mg/g) was found at 40°C temperature and 40% power. Following an analysis of the effects of continuous power (D: 70%) and temperature (A: 50°C), the highest value (1.265 mg/g) was obtained at 45 min and a solid/liquid ratio of 1:10. When the effects of constant temperature (A: 50°C) and solid/liquid ratio (C: 1/10) were examined, the maximum value (1.395 mg/g) was reached at 30 min and 100% power. Following an analysis of the effects of a constant temperature (A: 50°C) and duration (B: 45 min), the highest value (1.031 mg/g) was obtained at 1/10 solid/liquid ratio and 70% power.

The greatest value (48.84%) was achieved at 60°C for 60 min when the effects of a constant solid/liquid ratio (C: 1/10) and power (D: 70%) were examined in terms of antioxidant activity (Figure 4d). Investigating the effects of continuous time (B: 45 min) and power (D: 70%) yielded the highest value (54.49%) at 60°C and a 1/5 solid/liquid ratio. Considering the impacts of constant time (B: 45 min) and the solid/liquid ratio (C: 1/10), the maximum value (52.07%) was reached at 60°C temperature and 40% power. Considering how power (D: 70%) and temperature (A: 50°C) affect, the maximum value (55.51%) was reached at 60 min and a 1:15 solid/liquid ratio. The greatest value (52.83%) was obtained at 30 min and 100% power when the effects of a constant temperature (A: 50°C) and a solid/liquid ratio (C: 1/10) were examined. With constant temperature (A: 50°C) and duration (B: 45 min) considered, the highest result (51.01%) was reached at 1/15 solid/liquid ratio and 40% power.

Regression analysis results in the following second‐degree polynomial equations that are used to estimate the levels of total polyphenol, total flavonoid, total anthocyanin, and antioxidant activity. The equations use the following symbols: A for temperature (°C), B for time (minute), C for the solid/liquid ratio (grams per milliliter), and D for power (%):

Total polyphenolic content mg GAE/g extract=+7.470.36250.81500.76000.61500.04751.360.96251.601.330.04500.83830.23791.250.68540.00000.00000.00000.00000.33751.540.62750.88500.11750.00002.620.78500.98500.00000.00000.00000.00000.00000.00000.0000−A−B+C−D+AB−AC+AD+BC−BD−CD−A2+B2+C2+D2+ABC+ABD+ACD+BCD+A2B−A2C+A2D+AB2−AC2+AD2−B2C+B2D−BC2+BD2+C2D+CD2+A3+B3+C3+D3,Total flavonoid content mg QE/g extract=+0.83800.06530.01530.00550.08050.04200.00750.05280.01870.02320.04150.04880.02650.02510.03320.00000.00000.00000.00000.01720.01950.08130.02830.05420.00000.01180.00230.00550.00000.00000.00000.00000.00000.00000.0000+A+B−C+D+AB−AC+AD+BC+BD−CD−A2−B2−C2−D2+ABC+ABD+ACD+BCD−A2B+A2C−A2D+AB2−AC2+AD2+B2C+B2D+BC2+BD2+C2D+CD2+A3+B3+C3+D3,Total anthocyanin content mg C3G/g extract=+1.130.82880.20700.35950.14350.14470.49280.38820.01600.34550.13100.19920.09160.13930.28080.00000.00000.00000.00000.61580.63830.55881.110.87500.00000.54650.04300.23550.00000.00000.00000.00000.00000.00000.0000−A−B+C+D+AB−AC+AD+BC−BD−CD+A2−B2−C2−D2+ABC+ABD+ACD+BCD+A2B−A2C−A2D+AB2+AC2+AD2−B2C−B2D+BC2+BD2+C2D+CD2+A3+B3+C3+D3,Antioxidant activity %inhibition=+34.149.944.864.841.073.714.552.870.41754.281.830.33927.594.905.230.00000.00000.00000.00009.490.70502.725.213.790.00000.05253.2610.540.00000.00000.00000.00000.00000.00000.0000+A−B+C+D−AB−AC−AD+BC+BD−CD+A2+B2+C2+D2+ABC+ABD+ACD+BCD+A2B−A2C−A2D−AB2+AC2+AD2+B2C+B2D+BC2+BD2+C2D+CD2+A3+B3+C3+D3.

To confirm the model’s predictive ability, a final UAE was conducted under ideal circumstances in addition to the experiments. The optimal extraction parameters for the maximum biological activity values and extraction efficiency in the UAE were determined to be 60°C, 59.981 min, 1/5.258 g/mL solid/liquid ratio, and 100% power, in accordance with the Box–Behnken experimental design. These values led to the following results: 74.907% inhibition, 54% extraction efficiency, 0.878 mg QE/g, 3.607 mg C3G/g, and 11.233 mg GAE/g. As a result, 60°C, 60 min, 1/5 g/mL solid/liquid ratio, and 100% power extraction were the most closely matched experimental working conditions. With 10.67 mg GAE/g, 0.861 mg QE/g, and 2.169 mg C3G/g, 65.261% inhibition was attained. Consequently, the optimal extraction parameters for the UAE were determined to be 60°C, 60 min, 1/5 g/mL solid/liquid ratio, and 100% power. The findings supported the suitability of the established models and the experiments’ adherence to the ideal stage by demonstrating that a substantial difference (*p* > 0.05) between the experimental and predicted values was obtained.

Studies on the biological activity of Goji berries may show significant differences depending on the extraction method used, solvent type, plant species (such as red *L. barbarum* L. or black *L. ruthenicum* Murr.), geographical origin, harvest time, and the concentration of the extract being analyzed [38]. Both temperature and power are important parameters in enhancing the antioxidant activity of Goji berry extracts. Temperature increases the solubility and diffusion of bioactive compounds, while ultrasonic power accelerates the disruption of cell structures, thereby enhancing extraction efficiency. Determining optimal conditions (temperature, duration, solid/liquid ratio, and power) is critical for obtaining extracts with maximum antioxidant activity from Goji berries. The biological activity profiles of Goji berries obtained using UAE are generally consistent with results reported in the literature. In studies where UAE optimization was performed on Goji berries (*Lycium* spp.) and similar fruits, it was emphasized that optimal conditions depend on the balance between temperature, time, and ultrasonic power. Specifically, the 30°C–60°C range and medium‐level power settings have enabled the high‐level extraction of phenolic compounds and antioxidant activity in many studies. However, reported optimums vary depending on the matrix, solvent system, and response variable; therefore, when making direct numerical comparisons, methodological and scientific differences must be considered [18].

The cavitation effect is the fundamental mechanism of ultrasonic energy. In liquids, this effect leads to the formation, growth, and quick rupture of microscopic bubbles. Plant cell walls are physically and mechanically damaged by these explosions, which facilitates the quicker and more effective transfer of bioactive substances from the cell interior to the solvent [15]. Antioxidant activity is directly increased by this mechanism, which also directly impacts extraction efficiency [25]. Research generally confirms that increasing power and temperature within a suitable range tends to increase extraction efficiency and antioxidant activity. However, too much power can break down polysaccharide chains or cause compounds to degrade chemically [13].

Temperature accelerates mass transfer during extraction processes by improving the solvent’s penetration of plant material and the solubility of bioactive compounds. Antioxidant activity tends to rise when power and temperature are increased within a suitable range. Thermally unstable substances like phenolics and anthocyanins, however, may also degrade or undergo chemical changes as a result of extended exposure to high temperatures. Accordingly, it might result in a reduction of cytotoxic activity (as well as related antioxidant activity) [13, 31]. The extraction time gives the target compounds enough time to dissolve and diffuse. In general, longer times allow more compounds to enter the extract. However, excessively long extraction times can reduce yield by thermally degrading bioactive compounds, especially by breaking the chain structure of polysaccharides [37].

Acoustic cavitation causes increased mass transfer, which enables the solvent to enter the target compounds more rapidly and deeply. This contributes to the efficiency of ultrasonic extraction. Nevertheless, because of the high temperatures and locally formed reactive species (like hydroxyl radicals), high power or lengthy extraction times may cause bioactive compounds to degrade. In the same way, raising temperature and power initially increases extraction efficiency, but going over a certain point may cause degradation and a drop‐in bioactivity. This aligns with certain patterns of interaction found in the study (e.g., AD, AC, and BD) [39, 40]. Optimization studies in the literature generally show that antioxidant activity and total polyphenolic compound content may not always be directly correlated. In some cases, lower temperatures and shorter extraction times can preserve antioxidant activity, while higher temperatures can increase total polyphenolic compound content [15]. This is due to the differing thermal stabilities of phenolic compounds. The pattern observed in this study, where “some interactions are significant while some main effects are insignificant on their own,” is consistent with RSM‐based optimization results reported in the literature.

### 3.4. Rutin in *L. barbarum* Extracts Obtained Under Ideal Conditions: An HPLC Analysis

**Figure 5:**
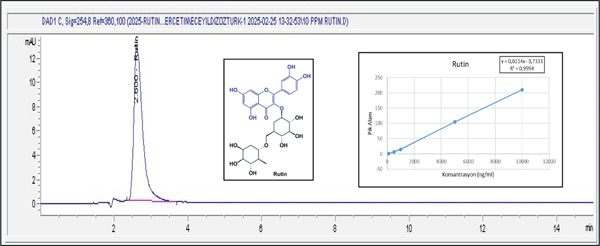
HPLC chromatogram of Rutin standard.

The presence of rutin in the extracts obtained under ideal conditions was discovered by HPLC analysis, and the results are shown. The reason for focusing on rutin in HPLC analysis in this study is that it is defined as a bioactive marker for Goji berries according to the European Pharmacopoeia [41] and is one of the most abundant flavonoids in Goji berries [21, 31] and because this compound is a primary focus in analytical and standardization studies. The standard rutin chromatogram is shown in Figure 5. The chromatograms of *L. barbarum* extracts made using various extraction methods and under ideal conditions are shown in Figure 6. Natural substances called rutin and quercetin, which are members of the flavonoid class, are well‐known for their immune‐boosting, anti‐inflammatory, and antioxidant qualities. Rutin is quercetin’s glycoside form, meaning that it is composed of sugar units like glucose and rhamnose. As a result, it becomes more soluble in water and breaks down in the intestines, releasing quercetin. When absorbed directly, quercetin, the pure flavonoid form, exhibits potent antioxidant properties despite having a lower bioavailability [42]. Rutin can therefore be readily dissolved using aqueous extractions and may be more beneficial in extract‐based formulations.

Figure 6HPLC chromatograms of *L. barbarum* extracts obtained under optimum conditions with different extraction techniques. (a) PWE extracts obtained at 50°C, 200 bar, and 30 min. (b) PWE extracts obtained at 60°C and 30 min. (c) MAE extracts obtained at 60°C, 45 min, 1/5 substrate/solvent ratio, and 900 W. (d) UAE extracts obtained at 60°C, 60 min, 1:5 g/mL substrate/solvent ratio, 100% power, and 80 kHz.

**Figure:**
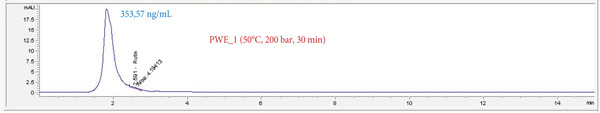
(a)

**Figure:**
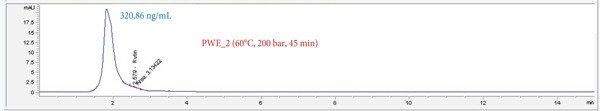
(b)

**Figure:**
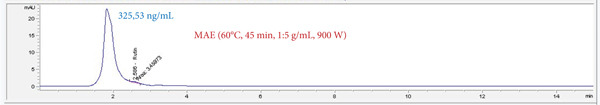
(c)

**Figure:**
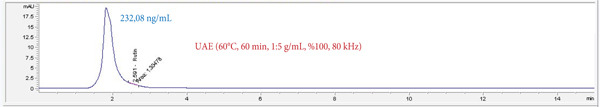
(d)

PWE, an environmentally friendly process that does not use hazardous solvents, produced the highest rutin content. This implies that phenolic compounds can dissolve more effectively due to the water’s decreased polarity at high pressures. By altering the dielectric constant of water and decreasing its polarity, this technique, which was performed at moderate temperatures of 50°C–60°C and high pressure (200 bar), enhanced the solubility and mass transfer of polyphenols [43]. This alteration makes it possible for water to more efficiently dissolve a greater variety of substances, especially those that are typically insoluble in water. This can improve phenolic compounds like rutin’s solubility and, as a result, their recovery [26]. The heat‐sensitive structure of rutin flavonoid was preserved, and a high yield of 0.35 mg/g was obtained at 50°C and 30 min. The low temperature and high pressure prevented rutin from degrading thermally. In contrast, the MAE and UAE methods’ ideal temperature of 60°C and high pressure of 200 bar partially degraded rutin’s thermal properties and decreased its effectiveness (Table 7, Figure 6). Being a water‐based system, PWE offers renewable solvent use when analyzed in terms of green chemistry. There is no solvent waste, making it eco‐friendly. Operating at low temperatures results in low energy consumption [44–46]. As a highly effective and environmentally friendly technique, PWE maintains the stability of flavonoids. It is the method that works best for regular flavonoids. It is safe to use in cosmetic and culinary applications.

**Table 7:** The amounts of rutin in *L. barbarum* extracts obtained under optimum conditions with different extraction techniques.

**Extraction Technique**	**Extraction Conditions**	**Rutin ng/ml (ppb)**	**Rutin (mg/g)**
**Pressurized Water Extraction (PWE)**	200 bar, 50^o^C, 30 min	353,57	0,35
**Pressurized Water Extraction (PWE)**	200 bar, 60^o^C, 45 min	320,86	0,32
**Microwave-Assisted Extraction (MAE)**	60^o^C, 45 min, 1:5 g/mL, 900 W	325,53	0,33
**Ultrasonically-Assisted Extraction (UAE)**	60^o^C, 60 min, 1:5 g/mL, 100%, 80 kHz	232,08	0,23

MAE is a technique that uses microwave energy to speed up extraction. In the solvent and plant matrix, microwaves directly transform electromagnetic energy into thermal energy. The release of bioactive compounds into the solvent is facilitated by the rapid breakdown of plant cell walls and increased molecular mobility caused by this dielectric heating. Benefits of this method include short extraction times, high extraction efficiency, and low energy consumption. Compounds like rutin may be recovered more readily thanks to this quick heating and cell disruption process [32]. Microwave heating quickly dissolves polyphenols and breaks down cell walls. But because polyphenols—particularly rutin—are heat‐sensitive, precise power and temperature settings are needed. Although somewhat lower, the rutin content obtained after 45 min of treatment at 60°C and 900 W of power is comparable to that obtained under the same conditions at 60°C and 200 bar of pressure (Figure 6). Given its short heating time, the microwave device used in green chemistry offers energy efficiency. With less solvent use, it is eco‐friendly. Compounds that are heat‐sensitive, like rutin, need to be treated carefully. The moderate yields of rutin produced by the MAE method are quick and effective. Nevertheless, flavonoid loss may result from an increase in temperature or from an excessive amount of microwave power [47, 48]. It is still a technique that adheres to green extraction principles.

UAE is based on the phenomenon of cavitation caused by ultrasonic waves. Cavitation occurs when small bubbles form in a liquid and suddenly collapse. This collapse creates localized high temperatures, pressures, and powerful mechanical shear forces [15]. These forces facilitate deeper penetration of the solvent into plant cellular materials, disrupt plant cell walls, and promote the release of intracellular ingredients into the solvent, thereby accelerating mass transfer and generally increasing extraction efficiency [8]. However, long extraction times and/or excessive ultrasonic power can cause bioactive compounds to oxidize or degrade. Excessive ultrasonic application has been shown to reduce extraction yield and alter the chain structure of polysaccharides. Likewise, it has been noted that when extraction times surpass 25 min, other bioactive substances, like anthocyanins, may deteriorate [32, 37]. It is possible that regular flavonoid degradation or transformation took place in the system running at a frequency of 80 kHz with 100% power for 60 min (Figure 6). Many studies might consider this to be a “long” time, and it might have caused sensitive compounds like rutin to degrade [25]. Rutin can oxidize as a result of reactive oxygen species (ROS) produced by excessive cavitation [2]. Although flavonoids typically have antioxidant qualities, they can oxidize and break down when exposed to high concentrations of ROS. High levels of ultrasonic stress have also been shown to excessively damage cell walls, which would decrease the amount of material that can be extracted [29]. Nevertheless, low solvent consumption offers an ecological benefit in terms of green chemistry. Thermodynamic equilibrium can be established since conditions that are nearly room temperature can be reached. Compared to MAE, less energy is used [15, 18, 49].

The yield obtained with UAE (0.23 mg/g) was found to be significantly lower than that obtained with PWE (0.35 mg/g) and MAE (0.33 mg/g) methods (Table 7, Figure 6). The yield from the UAE (0.23 mg/g) method was found to be substantially lower than the yield from the MAE (0.33 mg/g) and PWE (0.35 mg/g) methods. Acoustic cavitation that occurs during ultrasonic extraction could be the primary cause of this. Microbubble collapse during cavitation causes locally extremely high temperatures and pressures, increasing the risk of phenolic compound hydrolysis and thermal degradation [50, 51]. Under this abrupt energy intensity, flavonoid glycosides that are particularly sensitive to heat and oxidation, like rutin, may hydrolyze sugar bonds and transform into the aglycone form quercetin, or they may undergo oxidative degradation in their phenolic rings [52]. Furthermore, phenolic hydroxyl groups may be attacked by •OH and •H radicals that arise during cavitation, disrupting molecular integrity [53]. Additionally, compared to the solubility increase seen in PWE under high pressure due to reduced polarity, the dielectric constant of water as a solvent remains high under UAE conditions (60°C, atmospheric pressure), and its solubility capacity is more constrained [54]. On the other hand, by decreasing the polarity of water at high pressure (200 bar), the PWE technique improves the solubility and mass transfer of phenolic compounds. In addition, rutin’s thermal stability is maintained, and oxidative degradation is restricted at the relatively low temperature of 50°C [55]. As a result, PWE has produced the most rutin. In contrast, the MAE method uses microwave energy to quickly break down cell walls, which allows polyphenols to be released. Although there is a small chance that rutin will degrade during a 45‐min process at 60°C because of the higher temperature, the quick processing time and quick heat transfer guarantee that a high yield of 0.33 mg/g is maintained [56, 57].

In conclusion, dielectric heating, which is used in PWE and MAE, has the potential to improve routine recovery in the study by enhancing compound solubility and cell permeability. Cavitation in the UAE speeds up the extraction by breaking down cell walls, but if power and duration are not managed, it can result in a reduction in yield because it oxidizes and degrades sensitive compounds like rutin. Consequently, the ideal circumstances for every extraction method are crucial elements that influence the stability and recovery of particular bioactive substances. PWE is the technique that best preserves the stability of flavonoids; MAE offers the advantage of fast and effective extraction, while UAE shows lower performance in rutin yield due to structural degradation, radical oxidation, and solubility limitations caused by high‐intensity ultrasound. However, hybrid approaches incorporating additional target compounds alongside rutin may be evaluated in multicompound extraction strategies, as they are important for the recovery of total phenolics and anthocyanins.

### 3.5. Evaluation of the Bioactivities of Extracts From *L. barbarum* Obtained by Green Extraction Methods

In this study, three distinct green extraction techniques—PWE, MAE, and UAE—were compared to extract bioactive compounds from Goji berry fruit using only water as an eco‐friendly solvent. The extracts were then assessed for their antioxidant activity, phenolic compound, flavonoid, and anthocyanin content. According to the data gathered, every extraction technique has unique benefits with regard to particular bioactive substances and functional aspects.

The antioxidant qualities and polyphenol, flavonoid, and anthocyanin concentrations of the extracts made under optimal conditions were compared (Table 8). PWE has the greatest total phenolic concentration (17.753 mg GAE/g) when compared to other polyphenols. High pressure (200 bar) and controlled temperature (50°C) improved the solubility of polyphenols and increased the extraction efficiency of polyphenolic compounds by effectively dissolving the cell walls. The reason for this is that high pressure efficiently breaks down plant cell walls, allowing phenolic compounds to enter water [58]. This value was nearly reached by UAE (14.41 mg GAE/g), whereas MAE produced the lowest phenolic content (12.10 mg GAE/g). The extraction time and applied energy parameters were variable for both techniques, while the temperature remained constant at 60°C. Although some heat‐sensitive phenolics, like gallic acid, may be partially degraded, MAE is believed to provide more aggressive cell degradation in a short amount of time [13]. Nevertheless, PWE’s lower DPPH radical inhibition rate (48.089%) raises the possibility that the antioxidant capacity of the phenolics extracted using this technique is constrained.

**Table 8:** Comparison of biological activities of *L. barbarum* extracts obtained by various extraction processes under optimum conditions.

**Extraction Technique**	**Extraction Conditions**	**Total Phenolics** **(mg gallic acid/g extract)**	**Total Flavonoids** **(mg quercetin/g extract)**	**Total Anthocyanin** **(mg C3G/g extract)**	**Antioxidant Activity** **(% DPPH Inhibition)**
**Pressurized Water Extraction (PWE)**	200 bar, 50^o^C, 30 min	17,753	1,037	1,995	48,089
**Microwave-Assisted Extraction (MAE)**	60^o^C, 48 min, 1:5 g/mL, 900 Watt	12,10	1,077	1,197	75,942
**Ultrasonically-Assisted Extraction (UAE)**	60^o^C, 60 min, 1:5 g/mL, 100% Power	14,41	0,916	3,607	74,907

Abbreviations: DPPH, 2,2‐diphenyl‐1‐picrylhydrazyl radical scavenging activity; TAC, total anthocyanin content; TFC, total flavonoid content; TPC, total polyphenolic content.

When the obtained data were compared with the results in the literature, it was observed that the results were close or higher. Tripodo et al. (2018) extracted the phenolics from Goji berries using high‐pressure liquid extraction, yielding 5.72 mg GAE/G and 0.16 mg quercetin/g at 10 MPa pressure and 50°C. There were 8.31 and 28.8 mg GAE/g with 0.25 and 1.68 mg quercetin/g, respectively, when the temperature was raised to 115°C and 180°C. The optimal temperature was found to be 50°C because of the detrimental effects of high temperatures on anthocyanins, even if the amount of phenolic compounds rose with temperature [16]. Skenderidis et al. determined the total polyphenol content as 419.88 mg GAE/L in ultrasonic extraction applied at 57°C temperature and 221.72 W/cm^2^ ultrasonic power for 23 min [15].

The MAE yielded the highest value of 1.077 mg quercetin/g in terms of TFC. This outcome shows that microwave energy efficiently breaks down cell walls and is very effective at quickly and efficiently releasing flavonoids. The efficient breakdown of the cell wall and flavonoid resistance to microwave radiation are responsible for this outcome [47]. While the UAE method produced a lower yield of 0.916 mg QE/g, the PWE method produced a fairly comparable yield of 1.037 mg QE/g. The partial breakdown of flavonoids caused by the cavitation effect during ultrasound treatment explains this [15, 18].

One of the most striking results is that the UAE has the highest total anthocyanin concentration (3.607 mg C3G/g). This is because it is known that the cavitation produced by ultrasonic waves helps anthocyanins, in particular, dissolve intracellularly while maintaining their structural stability. Since the pigments are retained and extracted, this efficiency is increased by the UAE’s low temperature and high diffusion rates [18]. This could be because ultrasound waves selectively release compounds with a high pigment content. In anthocyanin extraction, the PWE (1.995 mg C3G/g) and MAE (1.197 mg C3G/g) approaches performed worse. This result implies that fragmentation may be caused by high temperatures and microwave energy, particularly in heat‐sensitive substances like anthocyanins [47, 48]. The results of the traditional solvent extraction study on Goji berry anthocyanin extraction in the literature are likewise noteworthy. Moura et al. found that a classical ethanol solvent extraction applied at 60°C for 60 min produced 4.17 mg/100 g of total anthocyanin and 0.326 mg/g of rutin [21]. The high extraction results in this study using green solvent water without harmful solvent residues demonstrate the promise of contemporary green techniques.

The MAE method yielded the highest DPPH free radical scavenging inhibition of 75.942%. This suggests that the extracted compounds have a high level of functionality and quantity of activity. With UAE, the DPPH inhibition rate was 74.907%, while with PWE, it was 48.089%. The improvement in free radical capture capacity and the change in phenolics’ isomeric structures may be the causes of MAE’s superiority [47]. It indicates that antioxidant activity is influenced not only by the overall phenolic content but also by the makeup of flavonoids and anthocyanins. By enhancing the stability of bioactive compounds, MAE may be thought to offer more potent DPPH free radical scavenging activity. Despite the high phenolic concentration of the extract, the decreased DPPH rate obtained with PWE may be due to a restriction of antioxidant activity caused by the high‐pressure effect or a variation in the chemical structures of the bioactive compounds [43]. PWE produced a 48.08% DPPH inhibition. Despite having a high phenolic yield, PWE raises the possibility that the compounds’ antioxidant potential is constrained. The absence of anthocyanins may be the cause of the comparatively low DPPH inhibition in spite of the phenolic content. Additionally, the structure, level of polymerization, and redox potential of the extracted phenolic compounds could be the cause of this discrepancy. It indicates that antioxidant activity is influenced not only by the overall phenolic content but also by the makeup of flavonoids and anthocyanins. Because microwave extraction increases the stability of bioactive compounds, it is believed to provide more effective radical scavenging activity [47, 48].

Beyond the research in the literature, which frequently concentrates on solvent‐based extractions, this study optimized green extraction techniques using only water‐based systems. Furthermore, a statistically sound comparison of the effectiveness of various technologies was made possible by parametric optimization employing RSM. Consequently, a scientifically grounded foundation for choosing water‐based extraction methods for industrial uses is provided. From an applied standpoint, the PWE method is a viable approach for producing high phenolic density nutraceutical raw materials in light of this information. Formulations of antioxidant supplements and functional food applications benefit from MAE’s high flavonoid content and antioxidant activity. It works well with fast‐acting supplements and functional drinks as well. Furthermore, the cavitation effect produced by ultrasound energy is especially effective in extracting pigment compounds with color (such as anthocyanins). For colored functional products (jelly beans, capsules, and functional drinks), this extract is particularly well suited. Its high anthocyanin content is linked to its antioxidant capacity, which is very similar to that of the microwave method. These results imply that the kind of bioactive compound being targeted should determine the extraction method to be used. Goji berry appears to be especially promising for use in functional food formulations when extracted using microwave and ultrasonic methods. The results will guide the development of new formulations for use in the phytotherapeutic, cosmetic, and functional food industries.

## 4. Conclusions

In this study, the methods of PWE, MAE, and UAE were compared and optimized to extract biologically active compounds from Goji berry fruits as efficiently as possible while preserving the environment. TPC, TFC, TAC, and DPPH inhibition values of the extracts produced under ideal conditions were assessed after the process parameters of all three methods were optimized using RSM. The results showed that bioavailable compounds from Goji berry fruit could be effectively extracted using all three extraction methods. PWE demonstrated its ability to resolve complex phenolic structures under high temperature and pressure by yielding the highest TPC (17.753 mg GAE/g extract) among the methods under investigation. Notwithstanding the benefits of PWE, it is essential to take into account any potential disadvantages, such as the possibility of sensitive compounds degrading thermally at high temperatures. Additionally, PWE equipment may require a larger initial setup cost than alternative techniques. Without optimization, its use on an industrial scale might be restricted due to energy consumption and equipment costs. On the other hand, MAE produced greater yields and the highest antioxidant activity (75.942% DPPH inhibition) in shorter amounts of time, which is most likely related to improved mass transfer and quick cell breakdown. MAE has strong antioxidant and flavonoid recovery properties, but it has a high energy cost and should be considered for the possibility of thermal degradation. The UAE demonstrated the highest anthocyanin extraction efficiency (3.607 mg C3G/g extract), confirming its applicability for the extraction of structurally sensitive compounds in mild heat environments. Maximum antioxidant efficiency and phenolic/anthocyanin recovery per energy are characteristics of the UAE. In terms of processing ease and energy cost balance, it is also the most environmentally friendly extraction technology.

There are distinct benefits to each extraction method for obtaining bioactive compounds from Goji berries. One of the most important criteria in food products is consumer safety and minimizing solvent toxicity. In this context, methods using food‐grade solvents such as water and ethanol should be preferred. For food applications, UAE, which is carefully optimized to protect heat‐sensitive compounds and adopt an environmentally friendly approach, may be preferred. This method is also advantageous in terms of energy cost balance. Exact control over time and power is essential for some compounds, like rutin, to prevent degradation. MAE can be a suitable substitute if high antioxidant activity and extraction rate are crucial. In the pharmaceutical industry, obtaining particular bioactive compounds at high concentrations and purity is usually the aim. If bioactive compounds are extracted from products with various pharmacological properties, such as anticancer, neuroprotective, anti‐inflammatory, and immunomodulatory effects, as found in Goji berries, cost may often be a secondary factor. PWE is a potent choice for pharmaceutical applications because it maximizes the total yield of polyphenolic compounds and yields a wide variety of compounds. In terms of time and efficiency in drug development processes, the high antioxidant activity and quick extraction time may be significant. MAE should be considered, especially in cases where specific compounds need to be obtained quickly and in high quantities. In both application areas, extraction conditions (temperature, time, solvent type and concentration, and power) must be carefully optimized according to the chemical structure and stability of the target compound to ensure the highest yield and biological activity.

The fundamental contribution to the literature is the comparative analysis of three distinct green extraction techniques (PWE, MAE, and UAE) for obtaining bioactive compounds from Goji berries. This comprehensively highlights the distinct benefits and drawbacks of each technique. The study’s findings give developers of functional food products a scientific and useful manual on which green extraction technique is best for optimizing a particular bioactive compound (anthocyanins for UAE) or a general bioactive property (antioxidant activity for MAE and TPC for PWE). This makes it possible for manufacturers to produce standardized, premium Goji‐based products with the intended health advantages. In conclusion, the research offers a thorough grasp of how well various green extraction techniques perform in terms of removing bioactive substances from Goji berries, providing crucial information for creative and sustainable product development in the pharmaceutical and functional food sectors.

In summary, this study shows that bioavailable compounds in functional foods like Goji berries can be obtained through the effective application of sustainable extraction technologies. The data collected is an essential starting point for the creation of green technologies that provide natural antioxidant sources for use in the food industry, as well as in cosmetic and pharmaceutical applications. Future research should concentrate on assessing these extracts’ bioavailability, shelf life, and effectiveness in functional product formulations. In order to optimize compound recovery and functionality, it should also concentrate on the bioaccessibility of the resultant compounds, the scalability potential of green extraction techniques, and synergistic multistep extraction strategies.

## Conflicts of Interest

The author declares no conflicts of interest.

## Author Contributions

All conceptualization, data curation, methodology, writing—original draft preparation, review and editing, visualization, interpretation of results, and supervision were performed by the author.

## Funding

This work was supported by the Yasar University Scientific Research Projects Office (BAP), Turkey, under Grant No. BAP‐139.

## Data Availability

The data that support the findings of this study are available from the corresponding author upon reasonable request.
